# Planarian regeneration in space: Persistent anatomical, behavioral, and bacteriological changes induced by space travel

**DOI:** 10.1002/reg2.79

**Published:** 2017-06-13

**Authors:** Junji Morokuma, Fallon Durant, Katherine B. Williams, Joshua M. Finkelstein, Douglas J. Blackiston, Twyman Clements, David W. Reed, Michael Roberts, Mahendra Jain, Kris Kimel, Sunia A. Trauger, Benjamin E. Wolfe, Michael Levin

**Affiliations:** ^1^ Allen Discovery Center at Tufts UniversityBiology DepartmentTufts University200 Boston Ave., Suite 4600MedfordMA02155‐4243USA; ^2^ Kentucky SpaceLLC, 200 West Vine St., Suite 420LexingtonKY40507USA; ^3^ NASA Kennedy Space CenterSpace Station Processing FacilityBuilding M7‐0360, Kennedy Space CenterFL32899USA; ^4^ Center for the Advancement of Science in Space (CASIS)6905 N. Wickham Rd., Suite 500MelbourneFL32940USA; ^5^ Exomedicine Institute200 West Vine St.LexingtonKY40507USA; ^6^ Harvard UniversitySmall Molecule Mass Spectrometry Facility52 Oxford St.CambridgeMA02138USA

**Keywords:** planaria, regeneration, space travel

## Abstract

Regeneration is regulated not only by chemical signals but also by physical processes, such as bioelectric gradients. How these may change in the absence of the normal gravitational and geomagnetic fields is largely unknown. Planarian flatworms were moved to the International Space Station for 5 weeks, immediately after removing their heads and tails. A control group in spring water remained on Earth. No manipulation of the planaria occurred while they were in orbit, and space‐exposed worms were returned to our laboratory for analysis. One animal out of 15 regenerated into a double‐headed phenotype—normally an extremely rare event. Remarkably, amputating this double‐headed worm again, in plain water, resulted again in the double‐headed phenotype. Moreover, even when tested 20 months after return to Earth, the space‐exposed worms displayed significant quantitative differences in behavior and microbiome composition. These observations may have implications for human and animal space travelers, but could also elucidate how microgravity and hypomagnetic environments could be used to trigger desired morphological, neurological, physiological, and bacteriomic changes for various regenerative and bioengineering applications.

## 1 INTRODUCTION

Planarian flatworms are known for their mastery of regeneration (Reddien & Sanchez Alvarado, 2004; Sanchez Alvarado, 2003; Sheiman & Kreshchenko, 2015). These bilaterians have the ability to completely recapitulate all body parts, including complex organs, from small pieces of the body, with high morphological and proportional fidelity (Hill & Petersen, 2015) in a vast variety of perturbations (Morgan, 1898). The complex organs include a full, centralized brain (Pagán, 2014; Sarnat, 1985) and central nervous system (Cebria, 2008) which has the ability to produce a continuous brain wave pattern (Aoki, Wake, Sasaki, & Agata, 2009) and complex behaviors (Corning, 1964; Inoue, Hoshino, Yamashita, Shimoyama, & Agata, 2015) with impressively variable sensory capabilities as inputs (Asano, Nakamura, Ishida, Azuma, & Shinozawa, 1998; Brown, 1962a, 1966; Brown & Park, 1964; Brown, Dustman, & Beck, 1966; Carpenter, Morita, & Best, 1974; Hyman, 1951; MacRae, 1967). Planaria exhibit complex learning, curiosity, and problem‐solving abilities (Best & Rubenstein, 1962; Corning & Freed, 1968; McConnell, 1965; Pagán, 2014; Wells, 1967). Moreover, they are able to repair and remodel three major polarity axes, dorsal/ventral, anterior/posterior, and medial/lateral, with outstanding accuracy (Gentile, Cebria, & Bartscherer, 2011; Gurley, Rink, & Alvarado, 2008; Kato, Orii, Watanabe, & Agata, 2001; Lange & Steele, 1978; Molina, Saló, & Cebrià, 2007; Orii & Watanabe, 2007; Owlarn & Bartscherer, 2016; Reddien, Bermange, Kicza, & Alvarado, 2007). These complex regenerative abilities are attractive for human regeneration research especially because planaria have more genomic similarities to vertebrates than do *Drosophila melanogaster* or *Caenorhabditis elegans* (Sánchez Alvarado, Newmark, Robb, & Juste, 2002). All of these properties make planaria a prime model for research in diverse areas of biomedicine, from stem cell biology to drug addiction (Rawls, Cavallo, Capasso, Ding, & Raffa, 2008a; Rawls, Gerber, Ding, Roth, & Raffa, 2008b; Rowlands & Pagan, 2008; Sacavage et al., 2008).

Patterning during regeneration, development, and cancer suppression is subject to the influence of physical forces including electric fields, magnetic fields, electromagnetic fields (Chernet & Levin, 2013; Funk & Monsees, 2006; Funk, Monsees, & Ozkucur, 2009), as well as other biophysical inputs (reviewed by Adams, 2008; Adams & Levin, 2013; Levin, 2014b; Lobikin, Chernet, Lobo, & Levin, 2012; Mustard & Levin, 2014; Stewart, Rojas‐Munoz, & Izpisua Belmonte, 2007). In planaria specifically, electric forces have been known to alter patterning information for decades (Bonaventure, 1957; Hyman, 1932; Lange & Steele, 1978; Marsh & Beams, 1952). More recently, bioelectric physiology has been implicated in the regulation of the cell cycle (Barghouth, Thiruvalluvan, & Oviedo, 2015), polarity (Beane, Morokuma, Adams, & Levin, 2011), and morphology (Beane, Morokuma, Lemire, & Levin, 2013; Emmons‐Bell et al., 2015) in the planarian as well. It is probable that physical forces, both internal and external, are modulated by the physical force of Earth's gravity, which probably influenced the way that the regenerative and developmental abilities of living organisms have evolved on Earth (Bizzarri & Cucina, 2014).

On Earth, biological systems are also subject to the naturally varying geomagnetic field (GMF) (Dubrov, 1978). This variation in geomagnetic disturbance has been shown to impact not only animal behavior (Beischer, 1971; Zamoshchina et al., 2012), but also medically relevant phenomena such as ciliary motion (Sandoze, Svanidze, & Didimova, 1995), stem cell function (Mo, Liu, Bartlett, & He, 2014), cardiovascular regulation (Cornelissen et al., 2001; Feigin et al., 2014; Gmitrov & Gmitrova, 2004; Stoupel, 2006; Stoupel et al., 2014), the autonomic nervous system (Baevsky, Petrov, & Chernikova, 1998), memory (Wang, Xu, Li, Li, & Jiang, 2003; Xiao, Wang, Xu, Jiang, & Li, 2009; Zhang et al., 2004), and the interactions between neurons (Shibib, Brock, & Gosztony, 1987). Magnetic field reversals may even have placed selective pressures on organisms that have contributed to subsequent extinction (Hays, 1971; Plotnick, 1980) and morphological change (Harrison & Funnel, 1964), and planaria have specifically been shown to be sensitive to weak magnetic fields (Brown, 1962b, 1966). These observations have been tested in recent decades by generating a near null or hypogeomagnetic field in order to understand the role of the Earth's natural magnetic field in numerous biological processes (Krylov, Bolotovskaya, & Osipova, 2013; Krylov et al., 2014; Zaporozhan, Nasibullin, Hozhenko, & Shapranov, 2002). The effects of exposure to a null magnetic field have included changes in immune response (Dorofteiu, Morariu, Marina, & Zirbo, 1995), axonal myelination (Shibib et al., 1987), and tubulin assembly (Wang, Wang, Xiao, Liu, & He, 2008), as well as developmental patterning (Asashima, Shimada, & Pfeiffer, 1991; Mo, Liu, Cooper, & He, 2012). The physiological mechanisms contributing to the influence of the GMF on biological events are currently unknown.

Biological systems also operate under the physical constraint of the Earth's gravity (Bizzarri, Cucina, Palombo, & Masiello, 2014). Therefore, an emergent question in recent years has concerned the behavior, cellular and otherwise, of organisms in microgravity conditions. It has since become clear that system level changes occur in microgravity fields (Crawford‐Young, 2003). More specifically, microgravity has been shown to affect cell morphology (Crawford‐Young, 2003; Testa et al., 2014), cytoskeletal organization (Masiello et al., 2014), early development (reviewed by Ogneva, 2015; see also Dournon, 2003), the likelihood of the open state of ion channels (Goldermann & Hanke, 2001), gene expression profiles (Pardo et al., 2005), differentiation (Pisanu et al., 2014), and apoptosis (Monici et al., 2006). Microgravity, in most cases so far, has been shown to be an inhibitor of tissue growth and regeneration in mammalian tissues (Blaber et al., 2014b). Microgravity research, on top of revealing how cells behave in response to altered physical forces, has also led to the development of innovative techniques. As an example, it has been found that 3D cultured cells allow for an unrestricted growth environment which is promising for the future of cell culture application to human medicine (Grimm et al., 2014; Souza et al., 2010).

Microbes are also impacted by space conditions. Classically, it was concluded that cells smaller than 10 μM, including bacteria, would be affected very minimally by weightlessness (Pollard, 1965); however, more recently, experiments observing microorganisms in space‐like environments have suggested otherwise (Horneck, Klaus, & Mancinelli, 2010). Moreover, microgravity conditions have been shown to increase bacterial growth kinetics, biofilm formation, and stress resistance (Kim, Matin, & Rhee, 2014; Rosenzweig et al., 2010). Microbes continue to maintain their adaptability in the changing environment and have been shown to change their secondary metabolite production, gene expression, and virulent capability (Leys, Hendrickx, De Boever, Baatout, & Mergeay, 2004; Nickerson et al., 2000, 2003). Although it still remains to be determined what physical factors are contributing to these changes (such as whether they are due to microgravity or fluid dynamics), it is clear that spaceflight can reshape microbial communities and what they produce. Aside from the clear biological implications, this also poses questions regarding manned spaceflight and protection from microorganisms that may be encountered while away from Earth.

If space travel environments can change cellular behavior and physiology, it is imperative to begin to understand how they can impact regeneration. Much of the previous work studying the impact of spaceflight on regeneration has been done in urodeles, in particular investigating limb and lens regeneration (Grigoryan, Mitashov, & Anton, 2002; Mitashov, Brushlinskaya, Grigoryan, Tuchkova, & Anton, 1996). Newts undergoing limb regeneration have shown increased regenerative rates on biosatellites as well as increased proliferation in limb blastemas in a synchronous manner. Lenses also showed increased regenerative ability. After landing, there was a two‐fold increase in the number of proliferative cells within the region that provides the cells for lens regeneration as well as other parts of the eye. Upon further investigation replicating these experiments in microgravity conditions on Earth, it was suggested that these effects occur due to weightlessness (Blaber, Sato, & Almeida, 2014a; Grigoryan, Anton, & Mitashov, 1998). Conversely, tail regeneration experiments did not find this same advancement in regenerative ability; however, changes in the pigmentation of tail blastemas in spaceflight animals were observed (Grinfeld, Foulquier, Mitashov, Bruchlinskaia, & Duprat, 1996). In *Schmidtea mediterranea* planaria, one study using simulated microgravity observed lethality while hypergravity led to decreased proliferation rates (Adell, Salo, van Loon, & Auletta, 2014). In contrast, another study found no distinguishing effects on *Girardia tigrina* (Gorgiladze, 2008). We used the species *Dugesia japonica*, not previously explored in space travel, with a range of analysis methods, to examine the effects of spaceflight conditions.

**Figure 1:**
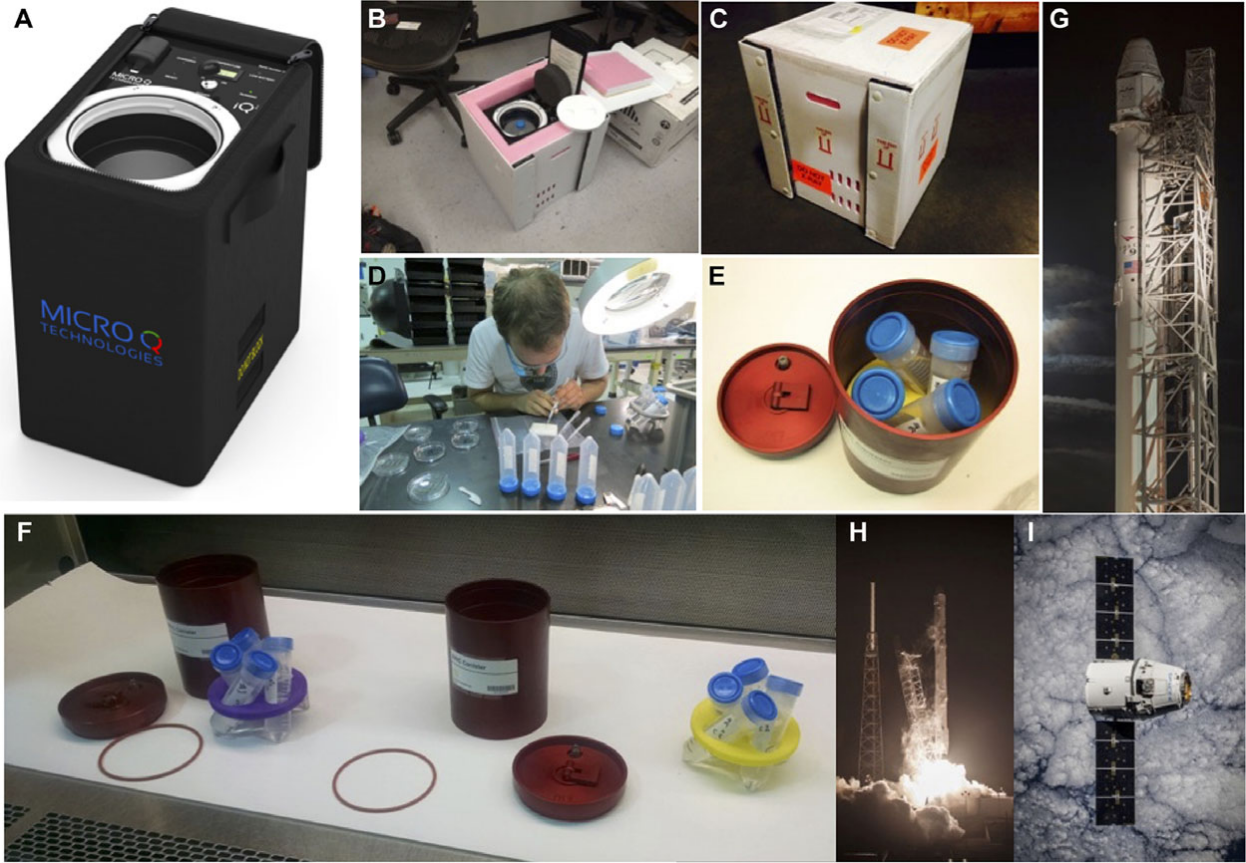
Pre‐launch preparation and logistics. For logistics on Earth, live worm samples were secured inside the battery powered refrigerated shipping container iQ2 from Micro Q Technologies (Scottsdale, AZ, U.S.A.), and FedEx Space Solutions (Memphis, TN, U.S.A.) was utilized for rapid shipment of the iQ2 container. (A) iQ2, the proprietary battery operated precision‐temperature‐controlled shipping container. (B), (C) iQ2 inside the protective shipping exterior. (D) Manual worm amputation at Kennedy Space Center prior to launch. (E), (F) 50 mL conical tubes (blue caps) containing live worms were sealed, then secured in 3D‐printed custom retainers (yellow and purple), and placed inside the BRIC‐100VC containers (red) provided by NASA. (G) SpX‐5 SpaceX Dragon Spacecraft on top of the Falcon 9 rocket at Cape Canaveral SLC‐40 launch pad. (H) SpX‐5 liftoff on 10 January 2015, at 09:47 UTC. (I) SpX‐5 SpaceX Dragon Spacecraft in orbit prior to berthing with the ISS on 12 January 2015. Images reprinted with permission from Micro Q Technologies (A) and of SpaceX (G–I)

Our study sought to determine how spaceflight and the conditions on the International Space Station (ISS) would affect planarian regeneration (Fig. 1). What effects would microgravity and micro‐geomagnetic fields produce, and might these effects be persistent after return to Earth? We used a panel of behavioral, microbiological, and morphological assays to understand how the total experience of spaceflight (including the stresses of take‐off and landing, as well as the weightless and 0 GMF conditions on the ISS itself) would affect this complex regenerative model system. This project was also designed to establish protocols for performing planarian research in space so as to determine proper transfer logistics and conditions for future missions. As humans transition towards becoming a space‐faring species, it is important that we deduce the impact of spaceflight on regenerative health for the sake of medicine and future space laboratory research.

## 2 RESULTS

Advances in regenerative medicine require an understanding of the remarkable mechanisms by which some organisms repair damage to their bodies. How these processes change when an organism is in outer space, in the absence of the normal gravitational and geomagnetic fields, is largely unknown. We undertook a series of experiments to understand the effects on organisms that spent an extended period of time in space. Planaria were either pre‐amputated or left as whole for spontaneous fission, and sealed into 50%/50% air/water tubes on Earth (Table 1). An identical set of worms were launched into space, spending over a month at the ISS under microgravity and micro‐geomagnetic force before returning to Earth. We evaluated these samples upon return, as well as after 20 months of maintenance in our laboratory; the latter time period was chosen as an optimal compromise between timely reporting of results so that they can contribute to the work of other groups and the ability to demonstrate truly long‐term consequencs of space travel.

**Table 1:** Initial number of worms per 50 mL tube, either whole or as amputated fragments

Tube number	Worm sample	Number of samples
1	Head (H)	15
2	Pharynx (P)	15
3	Tail (T)	15
4	Whole (W4)	4
5	Whole (W5)	5
6	Whole (W6)	6
7	Whole (W8)	8
8	Whole (W10)	10

### 2.1 Water shock

**Figure 2:**
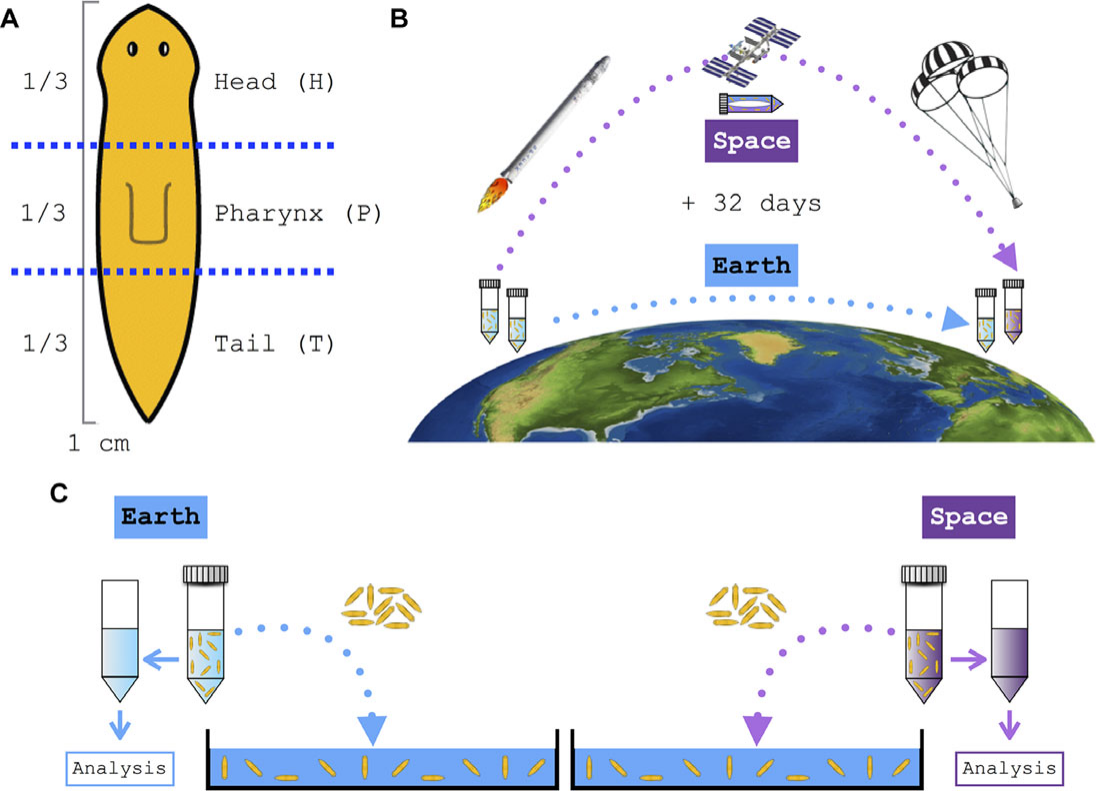
Flatworm amputation and space‐exposed and Earth‐bound worm sample schematics. (A) Approximately a third of the anterior part of the worm was cut off to create the head (H) fragment; then the posterior half was cut in half to create the pharynx (P) and tail (T) fragments, respectively. A total of 15 flatworms were cut and collected into three separate 50 mL conical tubes per fragment. (B) An identical number of worm samples, both whole and amputated fragments, were either sent into space or left on Earth for 32 days. (C) Immediately upon return to Earth, both space‐exposed and Earth‐only control worms from each sample tube were transferred to a Petri dish containing fresh Poland Spring water individually to identify any phenotypic changes

**Figure 3:**
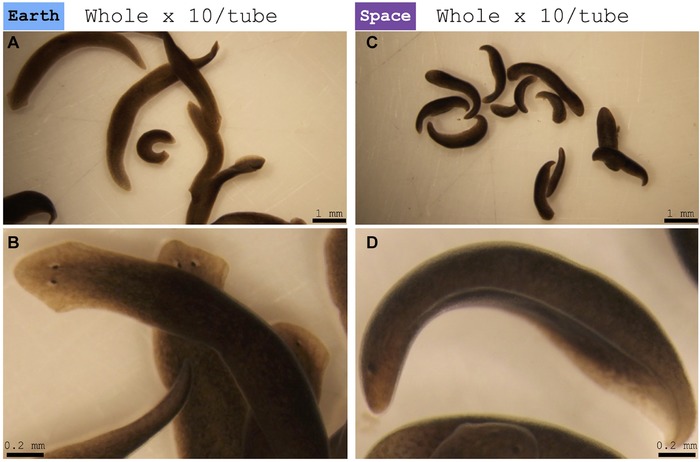
Water shock. (A), (B) Control worms left on Earth. (A) Representation of Earth‐only control worms, with full extension and rapid movement. (B) Close‐up image of representative Earth‐only control worm. (C), (D) Worms from space. (C) Representation of space‐exposed worms, in a state of water shock (ventrally curled and no movement). (D) Close‐up image of representative stocked space‐exposed worm (See also Videos S1 and S2.)

Immediately upon return to Earth, worms from each sample tube were transferred to a Petri dish containing fresh Poland Spring water to identify any phenotypic changes under the microscope (Fig. 2). The size of the worms did not differ appreciably between the two groups, within the normal variation of the length of *D. japonica* worms (data not shown). Surprisingly, only the sample containing 10 whole worms that had been launched into space showed immediate unusual behavior when introduced into fresh Poland Spring water: they curled up ventrally and were somewhat paralyzed and immobile (Fig. 3, and Videos S1 and S2). There was no sign of immediate blistering of the worm's epidermis, which generally indicates acute toxicity. This shock‐like phenotype lasted for an hour; the worms then gradually started to flatten out on the surface, slowly regaining movement, and after 2 h they all returned to normal behavior and morphology. This indicates that the sample of 10 whole worms that had been launched into space in a single sealed tube modified their biological state to accommodate the environmental change; when reintroduced into fresh water, the environmental change back to standard living conditions resulted in severe shock because of their altered metabolic state. Water shock was not seen in the later established ‘temperature‐matched’ Earth‐only control worms.

### 2.2 Fission in space

Whole worms sent into space were found to have fissioned spontaneously, but control whole worms on Earth had not (Table 2). Fission was not observed in either space‐exposed or Earth‐only worm fragments that had been manually amputated prior to launch (Table 3). It must be noted, however, that the control worms on Earth were kept at 20˚C at all times, while the worms in space unavoidably experienced somewhat higher temperatures at some time periods (Fig. S1). For this reason, the observed difference in spontaneous fission rate must be interpreted with caution.

**Table 2:** Number of whole worms before and after 1 month in a sealed tube, either while traveling to space and back or left on Earth. Worms which have traveled to space have shown spontaneous fissioning, while Earth‐only control samples have not. This is considered to be due to the slightly higher temperature the worms in space were maintained in during the mission

Start *n*	4	5	6	8	10
Earth	4	5	6	8	10
Space	7	7	10	14	13
Space/Earth	1.75×	1.4×	1.67×	1.75×	1.3×

**Table 3:** Number of manually amputated worm fragments before and after 1 month in a sealed tube, either while traveling to space and back or left on Earth. There is no difference in the resulting number of worms, indicating that no spontaneous fissioning has occurred during the mission. Note that pharynx fragments left on Earth did not survive the duration of the mission for an unknown reason

Fragment (*n*)	Head (15)	Pharynx (15)	Tail (15)
Earth	15	(−)	15
Space	15	15	15

### 2.3 Water composition

Samples of the water in which the space‐exposed worms and the Earth‐only worms had been living were frozen and stored at −20˚C. Although the water temperature of the worms in space was recorded throughout the mission, the information was not relayed in real time to Earth. After obtaining the space‐exposed worm water temperature information, a new set of ‘temperature‐matched’ Earth‐only control worms were kept in the same isolated conditions as before, with the temperature manually adjusted to follow the same profile and time course that the space‐exposed worms experienced.

Liquid chromatography–mass spectrometry (LC‐MS) analysis of the water samples revealed that both samples contained a large number of small organic molecules/metabolites. Whereas the total ion chromatograms of the two samples in the negative ion mode were similar (Fig. S2B), the total ion chromatograms of the two samples in the positive ion mode were quite different (Fig. S2A).

Our analysis of the unique ions observed in the space‐exposed worm water sample using the positive ion mode of LC‐MS indicate that many of them correspond to long‐chain fatty acids or mono‐hydroxylated/di‐hydroxylated long‐chain fatty acids. For example, a peak with an accurate *m*/*z* of 274.2730 is consistent with [M+NH_4_]^+^ of C_16_H_32_O_2_, which could be one or more skeletal isomers of hexadecanoic acid (e.g., CH_3_(CH_2_)_14_COOH or CH_3_(CH_2_)_7_CH[(CH_2_)_5_CH_3_]CO_2_H). Another peak, with an accurate *m*/*z* of 290.2680, is consistent with [M+NH_4_]^+^ of C_16_H_32_O_3_, which could be one or more regioisomers of hydroxyhexadecanoic acid [e.g., CH_3_(CH_2_)_13_CH(OH)CO_2_H or HO(CH_2_)_15_CO_2_H], the peak that has an accurate *m*/*z* of 334.2943 is consistent with [M+NH_4_]^+^ of C_18_H_36_O_4_, which could be one or more regioisomers of dihydroxyoctadecanoic acid [e.g., CH_3_(CH_2_)_14_CH(OH)CH(OH)CO_2_H or CH_3_CH_2_CH(OH)CH(OH)(CH_2_)_13_CO_2_H], and the peak that has an accurate *m*/*z* of 374.3617 is consistent with [M+NH_4_]^+^ of C_22_H_44_O_3_, which could be one or more regioisomers of hydroxydocosanoic acid [e.g., CH_3_(CH_2_)_19_CH(OH)CO_2_H or HO(CH_2_)_21_CO_2_H].

Our analysis of the unique ions from the space‐exposed worm water sample that were observed in negative ion mode indicates that molecules with long hydrocarbon chains were observed as well. For example, a peak with an accurate *m*/*z* of 285.2072 is consistent with [M−H^+^]^−^ of C_16_H_30_O_4_, which could be hexadecanedioic acid [HO_2_C(CH_2_)_14_CO_2_H], and a peak with an accurate *m*/*z* of 285.2072 is consistent with [M−H^+^]^−^ of C_19_H_37_NO_4_, which could be dodecanoylcarnitine, octanoylcarnitine *n*‐butyl ester, or *N*‐palmitoyl serine, all of which have long hydrocarbon chains.

LC‐MS/MS analysis revealed that the water samples contained several proteins. The list of identified proteins in the space‐exposed planaria water sample was filtered for reagents used in the trypsin digestion step, known contaminants (e.g., human keratin), and proteins that were also identified (i.e., the presence of at least one peptide in the mass spectrum) in the ground control sample. The remaining proteins were further filtered so that every protein on the list was identified via two or more unique peptides in the mass spectrum (Table S1).

Of the 11 proteins remaining on the list, we identified orthologs in *S. mediterranea* for nine of them (Table 4): we identified a homolog of fibrillin, a putative cathepsin C homolog, a putative myosin heavy chain homolog, a putative pyrophosphatase phosphodiesterase family member, a putative protease serine 12 neurotrypsin motopsin, a putative 14‐3‐3 protein, a putative tubulin beta homolog, a putative phosphoenolpyruvate carboxykinase, and a homolog of calmodulin. We conclude that exposure to space induces distinct differences in metabolism and/or secretion, which are detectable in the chemical composition of the animals’ milieu.

**Table 4:** Eleven proteins identified using mass spectrometry that were present in the water that housed the space‐exposed worms, but not the Earth‐only worms

Accession number of DNA sequence in *D. japonica*	Number of unique peptides found in mass spectrum	SmedGD ID of *S. mediterranea* homolog	Percent identity: *D. japonica* and *S. mediterranea*	Function in *S. mediterranea*
comp145442_c0_seq1	10	SMU15038343	73.13	Putative *C. elegans* protein MUA‐3 isoform 3 (fibrillin homolog)
comp128998_c0_seq1	3	SMU15000643	82.12	Putative cathepsin C
comp127150_c0_seq1	3	SMU15003136	86.87	Putative myosin heavy chain
comp131819_c0_seq1	2	SMU15000136	78.94	Putative pyrophosphatase phosphodiesterase family member
comp141188_c2_seq3	2	SMU15033813	68.08	Unknown function
comp145670_c2_seq3	2	SMU15005691	43.61	Putative protease serine 12 neurotrypsin motopsin
comp146596_c0_seq1	2	SMU15025275	93.36	Putative 14‐3‐3 protein
comp141858_c0_seq6	2	SMU15002375	97.52	Putative tubulin beta
comp135193_c0_seq1	2	SMU15002770	91.96	Putative phosphoenolpyruvate carboxykinase
comp86205_c0_seq1	2	SMU15039048	100.00	Putative protein CMD‐1 (calmodulin)
comp142073_c0_seq1	2	SMU15038409	53.64	Unknown function

### 2.4 Post‐space proliferation of the worm population

Worms that returned from space, together with the control worms on Earth, were then maintained separately in the laboratory under the same conditions, being fed organic calf liver paste every week for two additional months. After that time period, both populations were counted. We observed that the number of worms in the container that had gone to space was slightly less than the number of worms that remained on Earth (Fig. S3, Table S2). Likewise, in the worm fragments amputated prior to launch, the worm population exposed to space then grew more slowly than the Earth‐only controls.

### 2.5 Regenerative mispatterning

**Figure 4:**
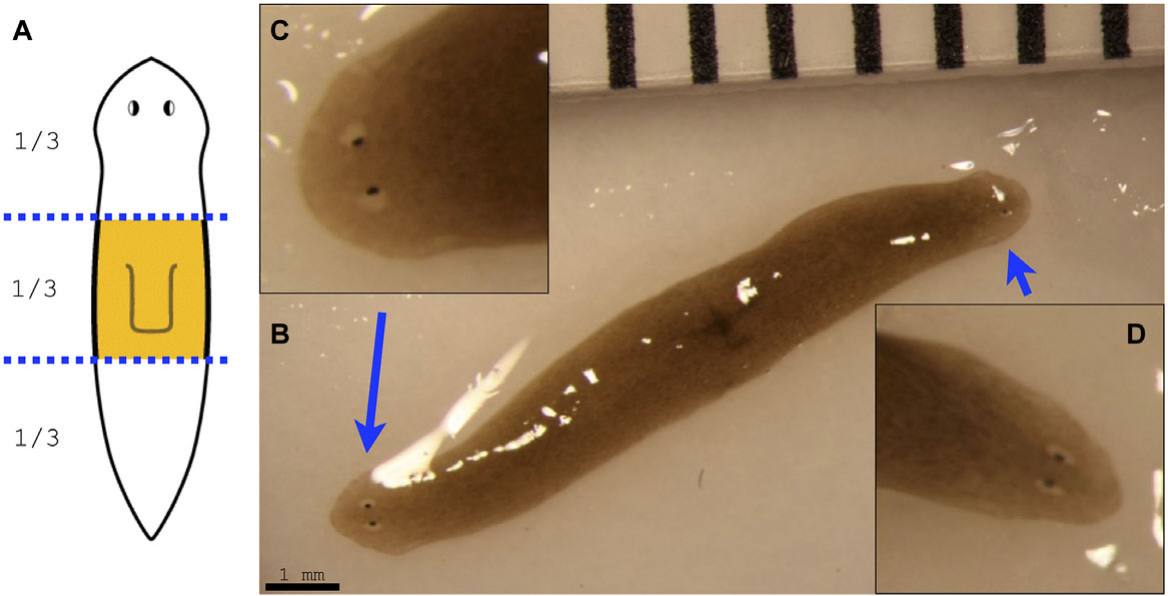
Double‐headed worm from space. (A) Schematic image of the original pharynx fragment, which traveled to space. (B) After return from space, one out of 15 pharynx fragments has regenerated into an extremely rare double‐headed worm. (C), (D) Close‐up images of each of the two regenerated heads

The most striking morphological change was observed with one of the 15 pharynx fragments from space which had been manually amputated on Earth prior to the launch. Figure 4 shows the unusual ‘double‐headed’ phenotype, which is extremely rare within a control population by spontaneous fissioning or even with manual amputation of a control worm. Although the sample number is low, the spontaneous occurrence of such a rare phenotype itself should be considered highly significant: in our own laboratory, we have not observed any spontaneous occurrences of double‐headedness in >18 person‐years of maintaining a colony of *D. japonica*. We estimate about 15,000 control worms in the last 5 years, without a single double‐headed animal arising from an untreated control fragment. Given this background, the *Z* score calculator for two population proportions gives a Z score of 31.6238 and a *p* value of <0.01 against chance.

**Figure 5:**
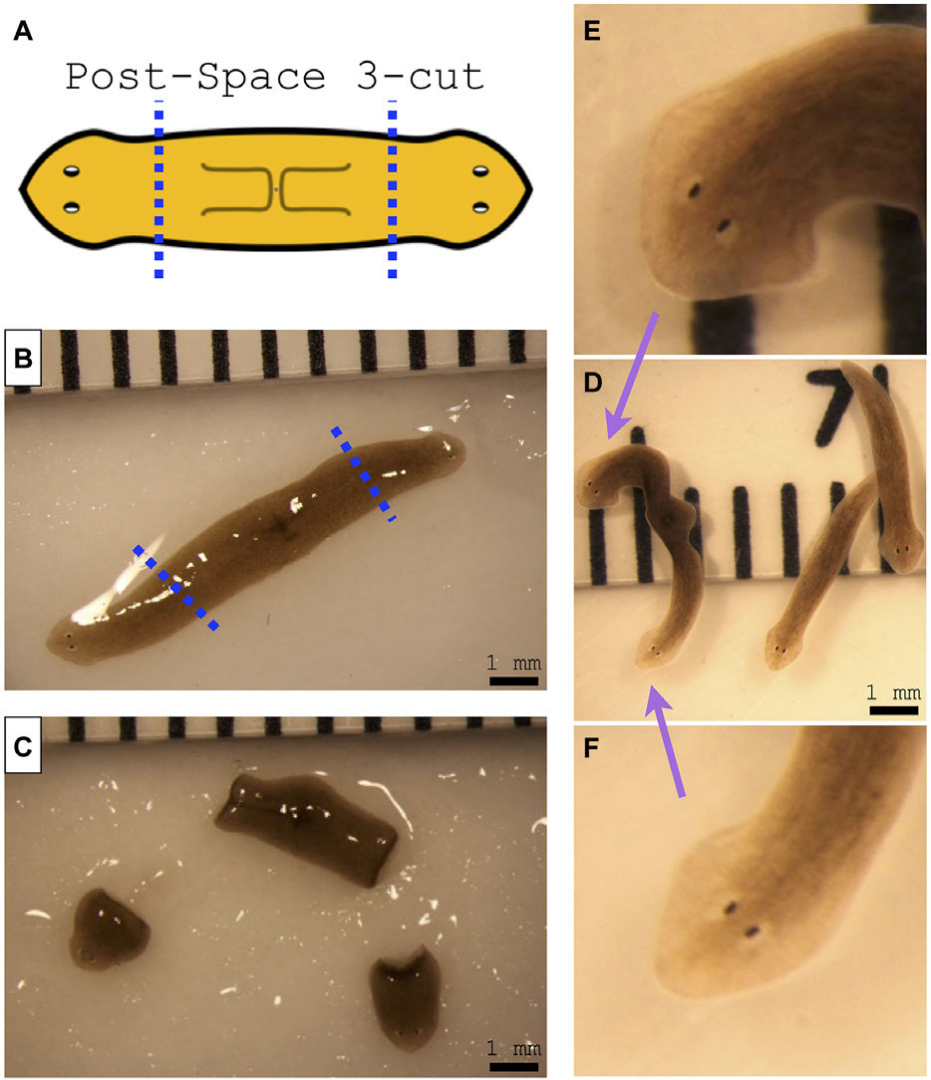
Amputation of double‐headed worm from space results in double‐headed morphology. (A) Schematics of amputation of the double‐headed space worm. (B) Double‐headed space worm before amputation at the dotted line; note that this photograph is the same as the image that appears in Figure 4B. (C) Double‐headed worm immediately after amputation of both heads. (D) Amputated double‐headed worm after 2 weeks of regeneration. Note that, while the two head fragments regenerated into two single‐headed worms like a normal worm, the head‐less fragment regenerated into a double‐headed worm. (E), (F) Close‐up images of each of the two regenerated heads of the re‐amputated double‐headed worm

We next amputated this specific double‐headed space‐exposed worm by making two decapitating cuts to remove both heads. Remarkably, the head‐less middle fragment regenerated into a double‐headed phenotype (Fig. 5), demonstrating that the major body‐plan modification that occurred in this animal is stable and persists for at least two rounds of cutting and subsequent regeneration after exposure to space travel. Given the long‐term alterations observed in these animals, we next asked whether two other aspects of their organismal physiology—behavior and microbiome composition—might also be permanently altered.

### 2.6 Behavioral alterations

**Figure 6:**
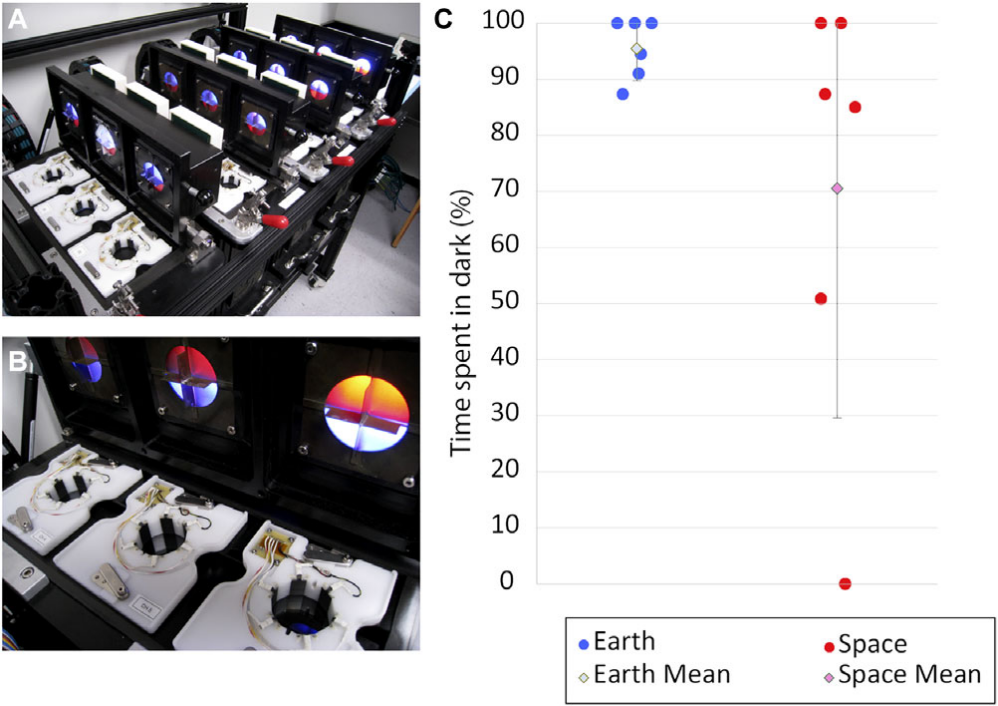
Space‐exposed worms demonstrate more variable photophobic behavior than Earth‐only worms. (A) Earth‐only and space‐exposed planaria were placed individually in an automated behavior device which recorded animal location, speed, and response to light. (B) Overhead illumination is provided by LEDs which illuminate half the arena with red light (invisible to planaria) and half with blue light. (C) Space‐exposed worms demonstrated significant variability in their photo‐aversive behavior compared to Earth‐only worms (*F* test, *p* < 0.001). *N* = 6 for both treatments. Error bars indicate ± 1 SD. In dot plots in (C), the lateral positioning of the dots, within each of the two groups, is only to enable the separate data points to be distinguished from each other even when they occupy the same horizontal coordinate

The behavior of space‐exposed and Earth‐only animals was tested in an automated assay 20 months after return to Earth. Individuals from each group were placed in individual arenas, illuminated half with red light (beyond the planarian visual spectrum) and half with blue light, for 18 h with lighting conditions reversing hourly (Fig. 6A, B). Movement rates for each individual were recorded across the trial using motion tracking cameras and background subtraction algorithms. There was no significant difference in the overall rate of motion between treatments (data not shown). We then scored the percentage of time each worm (of six, from control and space‐exposed groups) spent in the dark half of a Petri dish versus the blue light‐emitting diode (LED) illuminated half. The controls spent 95.5% of their time in the dark, as is normal for this negatively phototaxic species. In contrast, the worms that had experienced space travel spent only 70.5% of their time in the dark. While the difference in the two groups’ means was not statistically significant due to the small sample size (*t* test, *p* = 0.17), the variance was significantly different (*F* test, *p* < 0.001) between treatments: Figure 6C shows that the space‐exposed worms exhibited a much less uniform (i.e., more variable) preference for light levels (see also Table 5).

**Table 5:** Six worms from the Earth‐only and space‐exposed groups were tested for 10 min with respect to their positions in a Petri dish that was half dark and half lit up with blue LED light. The percentage of time each worm spent in the dark side of the dish was calculated by an automated machine vision system optimized for planaria

	Percentage of time spent in the dark quadrant over 10 min	
Worm no.	Earth‐only	Space‐exposed
1	87.3%	85.0%
2	91.0%	100.0%
3	100.0%	50.8%
4	100.0%	0.0%
5	94.5%	100.0%
6	100.0%	87.3%
Average	95.5%	70.5%

### 2.7 Culture‐based assessment of planarian microbiome

**Figure 7:**
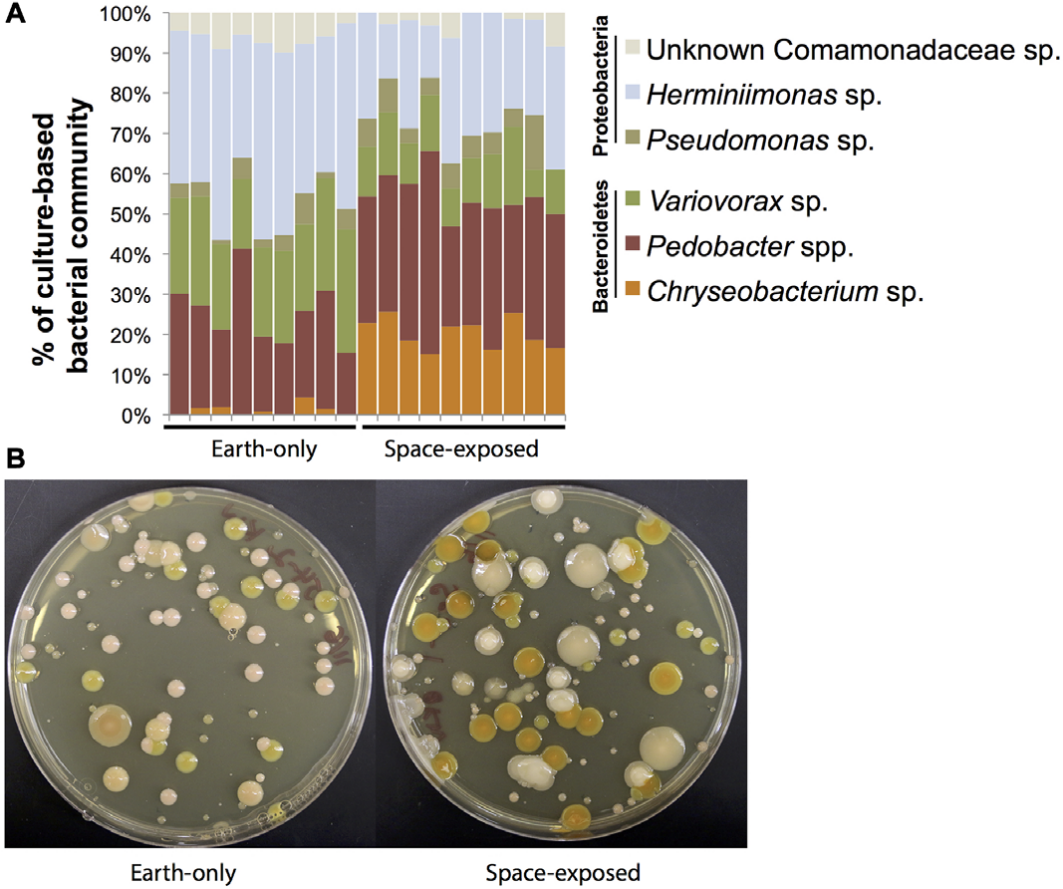
Bacterial community composition of Earth‐only and space‐exposed *D. japonica*. (A) Relative abundance of culture‐based morphotypes detected across Earth‐only (*n* = 9) and space‐exposed (*n* = 10) worms. (B) Representative plates showing bacterial morphotypes and distinguishable differences between space‐exposed and Earth‐only worms

Various genera of Proteobacteria (*Herminiimonas*, *Pseudomonas*, and an unknown bacterium in the family Comamonadaceae) and Bacteroidetes (*Chryseobacterium*, *Variovorax*, and *Pedobacter*) were the main bacterial morphotypes detected with culture‐based approaches (Fig. 7; Table 6). There was a significant difference in the composition of the culture‐based microbiome profiles between Earth‐only and space‐exposed worms (one‐way PERMANOVA *F* = 12.29, *p* < 0.001). The number of *Chryseobacterium* colonies significantly increased in space‐exposed worms, and *Variovorax*, *Herminiimonas*, and the unknown Comamonadaceae decreased in space‐exposed worms (*t* test, *p* < 0.01). We conclude that exposure to the conditions of space travel can alter bacterial community composition of *D. japonica*, and indeed does so in a manner that is still altered years afterward.

Taken together, our data reveal that exposure to space has very‐long‐lasting effects in this model organism, which include physiological, behavioral, morphological, and microbiological changes.

**Table 6:** Absolute bacterial densities (colony forming units per microliter of worm homogenate) across replicate Earth‐only (n = 9) and space‐exposed (n = 10) worms

	Earth‐only									Space‐exposed									
	1	2	3	4	5	6	7	8	9	1	2	3	4	5	6	7	8	9	10
*Chryseobacterium*	0	2	2	0	1	0	5	1	0	13	3.6	2	1.4	7	8	6	17	1.1	0.6
*Pedobacter*	34	29	19	31	20	27	25	20	6	18	4.8	4.2	4.7	8	11	13	18	2.1	1.2
*Variovorax*	27	31	21	13	24	35	25	19	12	7	2.2	1.1	1.3	3	4	5	13	0.4	0.4
*Pseudomonas*	4	4	1	4	2	6	9	1	2	4	1.2	0.4	0.4	2	2	2	3	0.8	0
*Herminiimonas*	43	42	47	23	53	69	43	23	18	15	1.9	2.9	1.2	10	11	11	15	1.4	1.1
Unknown Comamonadaceae	5	6	9	4	8	15	9	4	1	0	0.4	0.2	0.3	2	0	0	1	0.1	0.3

## 3 DISCUSSION

Our study examined how the regenerative and physiological properties of planaria changed during a space mission. Conditions associated with space are impossible to fully replicate on Earth, and yet must be explored due to the inevitability of the presence of humans and other organisms in space. We analyzed morphological, behavioral, bacteriological, and biochemical endpoints, finding not only a number of differences immediately after return to Earth, but also ones that persisted for 20 months. These are the first data exploiting a unique opportunity—exposing a highly tractable regenerative model system to space travel—which our laboratory will build on in future trips to the ISS.

This experiment faced a number of unavoidable limitations, some of which will be addressed in future missions. Maintaining the temperature of control worms on Earth exactly the same as those samples that traveled to space during the entire space mission was more challenging than anticipated. Future missions will achieve more consistent temperature control for the experimental samples, as well as provide real‐time data back to Earth which can be used to alter the temperature of Earth‐only controls in real time. The biggest unknown is likely to be stress associated with liftoff and splashdown, which cannot be easily replicated on Earth; future experiments will mimic this by applying similar mechanical disturbances of the Earth‐only control organisms. Thus, we do not individually implicate microgravity, vibration of liftoff, or 0 GMF in the effects we describe—the differences between space‐exposed and Earth‐bound controls are consequences of the entire process of delivery to, and return from, a space environment. It should be noted, however, that this is not simply a confounder: since any actual space travel will by necessity include all of these aspects, the effects must be studied as a real component of space travel which living systems will experience. Given the results reported from recent work (Adell et al., 2014) using much longer exposures to ***g*** forces of similar magnitude to that experienced by our samples during liftoff (∼3***g***) and landing (∼7***g***), we do not think it likely that our results are due to the brief periods of higher gravity that our planaria experienced. Future work will explicitly dissociate mechanistically the individual effects of the various stresses from the microgravity and micro‐geomagnetic force exposure per se.

The biggest factor reducing our ability to identify significant new regenerative phenotypes is probably the fact that worms were only put into space after being cut on Earth. We reduced the time between amputation and liftoff to the smallest delay compatible with the liftoff process, but it was not feasible to eliminate it completely due to the numerous logistics that have to take place before takeoff. Ideally, a forthcoming experiment will involve cutting them while in space; this experiment is important as many of the key steps of regeneration (and cellular decision‐making with respect to head−tail commitment of the blastema) occur very soon after cutting. In order for us to undertake this particular experiment, we will need to identify an astronaut residing on the ISS who is willing to assist us and is able to manually manipulate and cut the worms with a scalpel in microgravity. Future missions will also record ambient GMF values as a function of time throughout the experiment.

The finding of a single double‐headed worm in a population of 15 worms, which we have not observed in >18 person‐years of maintaining a colony of *D. japonica*, was exciting, even though it represents an *N* = 1 observation. Even more remarkable is the persistence of the phenotype, which recurred following a second and third round of amputation of the worm in normal conditions on Earth, in plain water, revealing a stable change to the organism's regenerative anatomy. It should be mentioned that recurrence of a two‐head phenotype in water‐only regeneration has been previously reported (Levin, 2014a; Oviedo et al., 2010); thus, while the reprogramming to a two‐head state was induced by space travel, its persistence across rounds of regenerations may be a general feature of such stable heteromorphoses (however induced) and not specifically due to space conditions.

While the exact mechanism of the induction of the two‐headed state by space travel is unknown, we can propose several hypotheses. It is known that reduced GMF disrupts cytoskeletal structures (Wang et al., 2008). It has also been shown that pharmacological disruption of microtubules induces double‐headed phenotypes (Mcwhinnie, 1955; Mcwhinnie & Gleason, 1957). Thus, one possibility is that the observed double‐headed worm was induced by a reduced GMF‐mediated disruption of cytoskeletal signaling. Another important recent finding is that microgravity alters ion channel electrophysiology (Richard et al., 2012); as several studies have shown the importance of endogenous bioelectrical signaling in regenerative patterning in planaria (Beane et al., 2011, 2013; Chan et al., 2014; Zhang, Chan, Nogi, & Marchant, 2011) and many other model systems (Bates, 2015; Levin, 2007, 2014b; Levin & Stevenson, 2012; Sundelacruz, Levin, & Kaplan, 2009), it is possible that some of our observed effects are mediated by alterations of ion channel function. Other possibilities include the effects of the space travel environment upon Wnt pathway molecules (Petersen & Reddien, 2007; Yazawa, Umesono, Hayashi, Tarui, & Agata, 2009) or physiological connectivity via gap junctions (Emmons‐Bell et al., 2015; Nogi & Levin, 2005; Oviedo et al., 2010). Especially interesting with respect to the hypothesis of gap junctional involvement is the recent observation that microgravity reduced the expression of two gap junction genes in embryonic stem cells (Blaber et al., 2015). Given the importance of gap junctions in planarian regeneration (Nogi & Levin, 2005; Oviedo et al., 2010) and in the control of stem cell (including planarian neoblast) biology (Oviedo & Levin, 2007; Oviedo et al., 2010; Starich, Hall, & Greenstein, 2014; Tazuke et al., 2002), this is a mechanism that will be investigated further in subsequent work.

Analysis using mass spectrometry revealed that the water samples contained many small molecules (Fig. S2) and several proteins (Tables 4 and S1). Additional analyses are needed to determine the exact molecular identity of the small organic molecules identified during the LC‐MS experiments and the reasons why (and the mechanism(s) by which) they are selectively produced by the space‐exposed planaria. It is interesting to note that others have reported that C16 and C18 fatty acids, like the ones identified in the positive ion mode of our LC‐MS experiment, can induce apoptosis (Ulloth, Casiano, & De Leon, 2003; Yan et al., 2016) and that hexadecanoic acid (also known as palmitic acid) can generate reactive oxygen species (Gao et al., 2014; Lambertucci et al., 2008). Since these worms, and not the Earth‐only control worms, experienced high ***g*** force during liftoff and landing, it is possible that the presence of these fatty acids directly caused cell death via apoptosis (leading to the release of cytoplasmic proteins discussed below) or were just released into the water from damaged tissue or ruptured cells on the surface of the worm.

Of the 11 proteins remaining on the list (Table 4) it was possible to find orthologs in *S. mediterranea* for nine of them, including a homolog of fibrillin, a putative cathepsin C homolog, a putative myosin heavy chain homolog, a putative tubulin beta homolog, and a homolog of calmodulin. While homologs of most of the proteins on this list are not known to be secreted into the extracellular medium (e.g., myosin, tubulin, and calmodulin), fibrillin (Jensen & Handford, 2016) and cathepsin C (Legowska et al., 2016) are bona fide extracellular proteins. How these proteins, or any of the proteins found on this list that are presumably intracellular proteins, ended up in the water that surrounded the space‐exposed worms is not yet clear. Since these worms, and not the Earth‐only control worms, experienced a high ***g*** force during liftoff and landing, it is possible that the proteins were released into the water from damaged tissue or ruptured cells on the surface of the worm. Alternative possibilities include novel secretion pathways activated by microgravity or altered GMF. Additional work is needed to determine what role, if any, these proteins play in the unusual ‘water shock’ behavior or in the behavioral experiments described earlier in this paper.

The worm microbiome was different between space‐exposed and Earth‐only worms (one‐way PERMANOVA *F* = 12.29, *p* < 0.001) (Fig. 7; Table 6). The density of *Chryseobacterium* colonies significantly increased in space‐exposed worms, and *Variovorax*, *Herminiimonas*, and the unknown Comamonadaceae decreased in space‐exposed worms (*t* test, *p* < 0.01). These shifts could be driven by the direct effects of microgravity on bacterial populations or indirect effects mediated through the planarian host. Bacteria and other microbes have recently been shown to impact the development of a variety of model organisms (Lee & Brey, 2013). There is limited work on planarian microbiomes, so it is currently difficult to know the causes and consequences of microbiome composition shifts for planarian regeneration and patterning. Recent work with the planarian *S. mediterranea* found similar bacterial types as we detected in *D. japonica*, and blooms of Proteobacteria in the *S. mediterranea* microbiome were associated with tissue degeneration, while high abundances of Bacteroidetes, including *Chryseobacterium* and *Pedobacter*, were associated with healthy animals (Arnold et al., 2016). Work is ongoing to analyze the functional significance of the *D. japonica* microbiome, but we predict that shifts in the ratio of Proteobacteria and Bacteroidetes may also impact *D. japonica* growth and development. *Chryseobacterium*, which was enriched in space‐exposed worms, is a widespread genus of bacteria, with some species being rare pathogens in humans (Mukerji, Kakarala, Smith, & Kusz, 2016) and others providing benefits to animal and plant hosts through improved growth and pathogen protection (Antwis, Preziosi, Harrison, & Garner, 2015; Coon, Vogel, Brown, & Strand, 2014). Future efforts will elucidate the functional consequences of *Chryseobacterium* and other *D. japonica* bacteria.

Our experiments illustrate a template for regeneration experiments in space, piloting many aspects of the crucial logistics of such research. Planaria are an excellent model for the investigation of physiology, host−microbe interactions, behavior, and anatomy of a complex species exposed to space travel. It is clear that exposure to these conditions induces a range of detectable and long‐lasting changes in these organisms. As spaceflight becomes more accessible, future work in this and other model organisms will surely uncover new details of the interactions between gravitational and geomagnetic fields and processes in living systems. Exciting opportunities for biomedical discoveries abound, not only in terms of learning to mitigate risk factors for human space travel, but also for the discovery of novel biophysical mechanisms that could be exploited both on Earth and in space in the regenerative medicine field. Finally, it should be pointed out that, in light of their remarkable self‐repair and complex behavioral capabilities, planaria themselves present an ideal design challenge for the next generation of space exploration robots.

## 4 MATERIALS AND METHODS

### 4.1 Animals

A clonal planarian flatworm stain of *D. japonica*, cultured at 20˚C in commercial Poland Spring water (Poland, ME, U.S.A.) in the dark, was used.

### 4.2 Logistics

As part of the SpaceX (Hawthorne, CA, U.S.A.) Commercial Resupply Service mission CRS‐5, also known as SpX‐5, live worms either whole or cut as indicated in Figures 1(D) and 2(A) were loaded into the SpaceX Dragon Spacecraft (Fig. 1G, I), which was launched into space on 10 January 2015 by the SpaceX Falcon 9 rocket from the Kennedy Space Center in Florida (Fig. 1H). The samples were exposed to the microgravity and micro‐geomagnetic field environment at the ISS approximately 78 h post‐amputation. The Dragon capsule returned to Earth into the Pacific Ocean off the coast of California approximately 32 days post‐amputation. Live worms were kept sealed inside 50 mL conical tubes and were secured inside the BRIC‐100VC (Biological Research in Canisters, see Fig. 1E, F) containers provided by NASA, with temperature data recorder attached, immediately prior to launch and throughout the mission.

Two sets of control worms were generated: one set of controls (‘concurrent’) consisted of live worms that had been sealed in Poland Spring water in the same manner as their space‐exposed counterparts and kept in full darkness at 20˚C for the same period of time as their space‐exposed counterparts. These worms were used as controls for all of the experiments described in this paper, except for the mass spectroscopy analysis. For the mass spectroscopy experiments, a second set of worms were generated after the space‐exposed worms returned to Earth that were ‘temperature‐matched’ (see Fig. S1), so that they experienced the same changes in temperature as the space‐exposed worms for the same duration.

For logistics on Earth between laboratories before launch and after splashdown, live worm samples were secured inside the proprietary battery operated precision‐temperature‐controlled shipping container iQ2 from Micro Q Technologies (Scottsdale, AZ, U.S.A., Fig. 1A–C), and FedEx Space Solutions (Memphis, TN, U.S.A.) was utilized for rapid shipment of the experimental worm group in the iQ2 container.

### 4.3 Air‐to‐water ratio in sample tubes

The initial CRS‐5 mission was expected to span a duration of approximately 30 days in space, starting from the Falcon 9 rocket launch out of Kennedy Space Center, the Dragon Spacecraft's docking with, berthed at, and then detaching from the ISS, until the Dragon capsule's return to Earth. The planarian worms were expected to survive within a sealed environment for a minimal 30‐day duration, without any water filtration/purification system. To determine the optimal air‐to‐water ratio of the sealed environment for a minimal 30‐day survival, 10 worms approximately 1 cm in length (average 0.25 g per worm) cultivated at 20˚C and starved for at least 1 week were sealed inside 50 mL conical tubes with different ratios of air to water and were maintained at either 10˚C or 20˚C in the dark. Aside from the worms with mostly no air, which survived for only 5 days, worms kept at 25%, 50%, or 75% air‐to‐water ratios survived for over 30 days, and up to 43 days after isolation. The water from the 75% air‐to‐water sample at 20˚C was transparent, but browner than water from the 25% air‐to‐water sample at 10˚C, which suggests that the water quality is declining more rapidly because of the higher air ratio and the higher culture temperature. This led us to determine the optimal condition for 30‐day survival of adding 25 mL of fresh Poland Spring water and air filling the rest of the 50 mL volume, at a 50% air‐to‐water ratio at 20˚C (to facilitate spontaneous fissioning while in space).

### 4.4 Membrane cap

In parallel, we also tested 50 mL conical tubes equipped with air‐permeable waterproof membrane caps, with the hope that improved gas exchange would lead to higher worm survival. Although all worms survived up to 49 days, interestingly the worms kept inside membrane cap tubes were outlasted by worms sealed in non‐membrane tubes, while losing a small amount of water along the duration possibly due to evaporation. We concluded that an optimal experimental 30‐day condition in space was a maximum of 10 worms (1 cm in length), sealed inside a non‐membrane 50 mL conical tube, with 50% air‐to‐water ratio, maintained at 20˚C (to facilitate spontaneous fissioning while in space).

### 4.5 Spontaneous fission

Since direct manipulation of the worms while in space was not an option for this mission, we next examined if this condition was suitable to facilitate spontaneous worm fissioning. We tested with different numbers of worms (4, 6, 8, or 10) sealed into a 50 mL conical tube with 50% air‐to‐water ratio, and this resulted in all worms fissioning within 1 week at 20˚C.

### 4.6 Pre‐launch amputation and preparation

In addition to having the worms spontaneously undergo fission and regeneration while in space, we also included worms amputated on Earth just prior to the launch. Fifteen flatworms in total were amputated by hand (Fig. 1D) on a stack of wetted filter paper using a scalpel into three different fragments (head, pharynx, tail; see Fig. 2A) on Earth approximately 31 h before launch. Fifteen of each fragment were separated and sealed into individual 50 mL conical tubes, with 50% air‐to‐water ratio. This is approximately equivalent to five whole worms per tube. Also, 4, 5, 6, 8, or 10 whole worms were also sealed into individual 50 mL conical tubes with 50% air‐to‐water ratio. A total of eight 50 mL conical tubes (Table 1) were then secured by custom 3D‐printed retainers inside two sealed BRIC‐100VC containers provided by NASA (Fig. 1E, F), with a temperature data logger secured inside each BRIC. As controls on Earth, eight tubes of exactly the same number of whole worms and amputated fragments were sealed inside 50 mL tubes with 50% air‐to‐water ratio and kept in the laboratory at 20˚C in the dark. These samples are considered concurrent (constant temperature) controls. The time from amputating the worms to the worms reaching the ISS was approximately 78 h, based on the temperature data retrieved from the attached data logger (see Fig. S1). The optimal temperature range for worms’ long‐term survival is considered a minimum of 10˚C to a maximum of 25˚C, with a short‐term durable range from 0˚C to 30˚C. The data logger indicates that the worms were maintained within the optimal temperature range throughout the mission (Fig. S1), with the help of sophisticated temperature‐maintaining gel packs provided by NASA during transport to and from the ISS in the Dragon Spaceship, similar to commercial ice packs, and while situated in an incubator onboard the ISS.

### 4.7 Post‐splashdown

The worm samples spent approximately 29 days at microgravity and in a micro‐geomagnetic field environment in the ISS. After return to Earth, the worm samples were received in our laboratory approximately 68 h after splashdown. The worms were immediately subjected to basic analysis in the laboratory. All space worms were alive inside the sealed tubes. Live worms were either photographed or had video movies taken with a Canon (Tokyo, Japan) Rebel T3i DSLR (digital single‐lens reflex) camera attached to a Zeiss (Oberkochen, Germany) Stemi V6 dissecting microscope. Water from the concurrent, Earth‐only control and space‐exposed worms was frozen immediately after return to Earth and stored at −20°C.

### 4.8 Mass spectrometry analysis: small molecules/metabolites

The water which the space‐exposed planaria and the Earth‐only planaria inhabited during the course of the experiment was thawed in cold water, and 4.0 mL aliquots of each sample were freeze‐dried using a lyophilizer. The samples were then re‐suspended in 100 μL of 60% acetonitrile. The sample was centrifuged at 14,000***g*** to remove debris. 5 μL of this reconstituted sample was injected for each LC‐MS analysis. A Thermo q‐Exactive Plus mass spectrometer (Thermo Fisher Scientific, Waltham, MA, U.S.A.) coupled to a Thermo Ultimate 3000 (Thermo Fisher Scientific) high performance liquid chromatograph (HPLC) was used to perform the LC‐MS analysis of metabolites in biological samples and authentic chemical standards in both positive and negative ion mode using polarity switching. Two separate data‐dependent MS/MS analyses were conducted in positive and negative ion mode using the top five ions using dynamic exclusion for 30 s. Electrospray source settings included a sheath gas flow rate set at 35 (arbitrary units), an auxiliary gas flow rate at 5 L/min, a capillary temperature of 250°C, and an auxiliary gas temperature of 300°C. A calibration of the *m*/*z* range used was performed using the Thermo LC‐MS calibration mix immediately prior to the analysis for both positive and negative ion mode. A scan range of 66.7−1000 *m*/*z* was used at a resolving power of 70,000 with alternating positive and negative ion mode scans. The chromatographic separation of metabolites was performed using hydrophillic interaction liquid chromatography (HILIC) on a SeQuant^®^ ZIC^®^‐pHILIC column, 5 μm, polymer PEEK 150 mm × 2.1 mm column (EMD Millipore, Billerica, MA, U.S.A.) at a flow rate of 0.1 mL/min. Mobile phase A was 20 mM ammonium bicarbonate with 0.1% ammonium hydroxide, and mobile phase B was acetonitrile. The mobile phase composition was started at 100% B and subsequently decreased to 40% B over 20 min. The column was then washed at 0% B for 5 min before re‐equilibration to 100% B over 15 min.

The space‐exposed planaria and the Earth‐only planaria samples were analyzed for differences using XCMS (Tautenhahn, Patti, Rinehart, & Siuzdak, 2012): significant differences between the two samples were observed in positive ion mode (Fig. S2A), but fewer differences were observed in negative ion mode (Fig. S2B).

### 4.9 Mass spectrometry analysis: proteins

The water in which the space‐exposed planaria and the later established ‘temperature‐matched’ Earth‐only control planaria inhabited during the course of the experiment was thawed in cold water, and 4.0 mL aliquots of each sample were freeze‐dried using a lyophilizer. The samples were then re‐suspended in 100 μL of tetraethylammonium bromide (TEAB), reduced with 20 mM tris‐(2‐carboxyethyl)phosphine (TCEP) in 25 mM TEAB at 37°C for 45 min, and alkylated with 10 mM iodoacetamide (Sigma) in 25 mM TEAB and kept in the dark, room temperature, for 45 min. Then 2 μg of trypsin/LysC (V5073, Promega, Fitchburg, WI, U.S.A.) was added overnight. 14 μL from a final volume of 140 μL was injected into the instrument after a hard spin and supernatant was removed to a new HPLC vial as there were particulates on the floor of each digestion tube. LC‐MS/MS was performed on an Orbitrap Fusion Lumos™ Tribrid™ (Thermo Fisher Scientific) mass spectrometer with the 100716L 90 min ID 150 nL KASIL trap 300 bar method to generate a list of proteins that were present in each of the samples.

Each sample was submitted for a single LC‐MS/MS experiment that was performed on an LTQ Orbitrap Elite (Thermo Fisher Scientific) equipped with a Waters (Milford, MA, U.S.A.) NanoAcquity HPLC pump. Peptides were separated onto a 100 μm inner diameter microcapillary trapping column packed first with approximately 5 cm of C18 Reprosil resin (5 μm, 100 Å, Dr Maisch GmbH, Germany) followed by an analytical column ∼20 cm of Reprosil resin (1.8 μm, 200 Å, Dr Maisch GmbH, Germany). Separation was achieved through applying a gradient from 5% to 27% acetonitrile in 0.1% formic acid over 90 min at 200 nL/min. Electrospray ionization was enabled through applying a voltage of 1.8 kV using a home‐made electrode junction at the end of the microcapillary column and sprayed from fused silica pico tips (New Objective, MA, U.S.A.). The LTQ Orbitrap Elite was operated in the data‐dependent mode for the mass spectrometry methods. The mass spectrometry survey scan was performed in the Orbitrap in the range 395–1800 *m*/*z* at a resolution of 6 × 10^4^, followed by selection of the 20 most intense ions (TOP20) for CID‐MS2 fragmentation in the ion trap using a precursor isolation width window of 2 *m*/*z*, automatic gain control (AGC) setting of 10,000, and a maximum ion accumulation of 200 ms. Singly charged ion species were not subjected to CID fragmentation. Normalized collision energy was set to 35 V and an activation time of 10 ms, AGC was set to 50,000, the maximum ion time was 200 ms. Ions in a 10 ppm *m*/*z* window around ions selected for MS2 were excluded from further selection for fragmentation for 60 s.

Raw data were submitted for analysis in Proteome Discoverer 2.1.0.81 (Thermo Fisher Scientific) software. Assignment of MS/MS spectra was performed using the Sequest HT algorithm by searching the data against a protein sequence database including all entries from the Human Uniprot database (SwissProt 16,768 and TrEMBL 62,460; a total of 79,228 protein forms, 2015) and other known contaminants such as human keratins and common laboratory contaminants. Sequest HT searches were performed using a 20 ppm precursor ion tolerance and requiring each peptides’ N/C termini to adhere with trypsin protease specificity while allowing up to two missed cleavages. Cysteine carbamidomethyl (+57.021) was set as static modifications while methionine oxidation (+15.99492 Da) was set as variable modification. An MS2 spectra assignment false discovery rate of 1% on protein level was achieved by applying the target‐decoy database search. Filtering was performed using a Percolator (64 bit version, reference 6). For quantification, a 0.02 *m*/*z* window was centered on the theoretical *m*/*z* value of each of the six reporter ions and the intensity of the signal closest to the theoretical *m*/*z* value was recorded. Reporter ion intensities were exported in the result file of Proteome Discoverer 2.1 search engine as Excel tables. All fold changes were analyzed after normalization between samples based on total unique peptide ion signal.

The list of identified ‘space worm’ proteins was filtered for reagents used in the trypsin digestion step, known contaminants (e.g., human keratin), and proteins that were also identified (i.e., the presence of at least one peptide in the mass spectrum) in the Earth‐only control sample. The remaining proteins were further filtered so that every protein on the list was identified via two or more unique peptides in the mass spectrum.

The full DNA sequences of the identified proteins were obtained from a recently published *D. japonica* transcriptome (Chan et al., 2016) and translated into potential protein sequences in all three reading frames using ExPASy (http://web.expasy.org/translate/). These *D. japonica* putative protein sequences (Table S1) were then subjected to a BLASTP search against the Smed_unigenes_20150217.aa database housed at the *S. mediterranea* Genome Database website (http://smedgd.stowers.org/) (Robb, Gotting, Ross, & Sanchez Alvarado, 2015). The resulting *S. mediterranea* ortholog was then subjected to pairwise BLASTP search against the sequence identified during the original mass spectrometry experiments to determine how similar the sequences of the *D. japonica* and the *S. mediterranea* proteins were (Supplemental Data and Table S1).

### 4.10 Analysis of planaria regeneration upon return to Earth

Live whole worms that returned from space were maintained in fresh Poland Spring water and kept at 20˚C in the dark, with weekly feeding of organic calf liver paste.

### 4.11 Behavioral analysis

Randomly chosen whole worms from the space‐exposed and Earth‐only colonies were tested, 20 months after return to Earth, in an automated behavior platform as described previously (Blackiston, Shomrat, Nicolas, Granata, & Levin, 2010; Shomrat & Levin, 2013). Briefly, the device consists of 12 individual arenas each containing a standard disposable 60 mm × 15 mm Petri dish filled with 15 mL of Poland Spring water. Above each arena, an illumination control head provides red or blue light independently, or in combination, to each quadrant of the dish in 12 even intensity steps via LED illumination (Opto Semiconductors GmbH, Regensburg, Germany: blue LED; 470 nm part no. LBW5SM, red LED, 635 nm part no. LRG6SP). Below each dish, a motion tracking camera (Insight‐Micro 1400, Cognex Corporation, Natick, MA, U.S.A.) records the position of each animal every 1000 ms via hardware background subtraction algorithms (this capture rate was chosen to minimize centroid bounce, which can artificially inflate movement rates of slow moving irregular objects). Planarian locations, and lighting conditions, are recorded as a log file, which can be analyzed to determine animal movement rates, color preference, preference for edge versus center of the arena, and rotation direction (clockwise vs. counterclockwise).

The behavior trial lasted 18 h and consisted of the following settings. Background illumination in all quadrants was 50 lm red light, which is undetectable to planarians given their photoreceptor profiles (Azuma, Iwasaki, & Ohtsu, 1999; Brown & Ogden, 1968). In addition, half of the arena is illuminated by 420 lm blue light, giving the animals a choice between “light” and “dark” halves of the environment. Every hour, the lighting conditions were inverted, causing animals in the dark portion of the arena to be exposed to light and vice versa. Light rotation served dual roles. First, as planaria are photophobic it promoted movement of animals across the trail, and allowed movement rates to be compared across treatments. Second, without rotation, animals that remained stationary during the course of the experiment would be scored as having a 100% preference for either light or dark, depending on their starting position. By inverting the light, stationary behavior would instead result in a 50/50 preference, and would thus not bias the averages towards either extreme.

### 4.12 Culture‐based assessment of planarian microbiome

To determine differences in bacterial community composition between space‐exposed and Earth‐only worms, we used culture‐based assessments of individual worm microbiomes. We chose to use culture‐based approaches because unpublished work from our laboratories and recent studies of the *S. mediterranea* microbiome (Arnold et al., 2016) suggest that dominant bacteria types in planarian microbiomes are culturable, and bacterial colony morphotypes of dominant bacterial genera can be easily distinguished.

Ten randomly chosen worms from each group were homogenized in 400 μL of phosphate‐buffered saline using a sterile micropestle. Homogenates were serially diluted in phosphate‐buffered saline and plated on brain heart infusion agar plates and were incubated for a week at 24°C. Representative morphotypes that grew were identified based on previous in‐house sequencing and identification of *D. japonica* bacterial isolates. To confirm the identities of these morphotypes, representative colonies were isolated, DNA was extracted using a PowerSoil® DNA Isolation Kit (MO BIO Laboratories, Carlsbad, CA, U.S.A.), and the the 16S rRNA region was amplified using the polymerase chain reaction primers 27f‐1492r; resulting amplicons were sequenced using Sanger sequencing. Differences in total community composition for the culture‐based microbiome data were determined using PERMANOVA, and differences in relative abundances of individual bacterial groups were determined using *t* tests, with Bonferroni corrections for multiple comparisons.

## Supporting information

Figure S1. Temperature profile for planaria during the SpX‐5 mission. The timing of critical events is also listed below the plot.

Figure S2. Total ion chromatograms for Earth‐only (red) and space‐exposed (blue) worms. (A) The LC‐MS was run in positive ion mode. (B) The LC‐MS was run in negative ion mode.

Supplemental Figure 3. Worm colony growth before and after being in space. The number of worms before and after one month in a sealed tube, either while traveling to space and back (circles + dotted lines), or left on Earth (triangles + solid lines), together with number of worms after additional two months on Earth (same starting sample N = same color). Worm colonies which have traveled to space, all have shown slightly reduced rate in colony size growth, compared to their Earth counterparts. (See Supplemental Table 2.) Note that the colony from 10 whole worms left of Earth did not survive the duration of the additional two months on Earth for an unknown reason.

Supplemental Data: Protein sequence alignments for the proteins identified in Supplemental Table 1

Table S1: Proteins identified using mass spectrometry that were present in the space‐exposed worm water sample but not in the Earth‐only worm water sample

Supplemental Table 2: Worm colony growth after space mission.

Video S1. Control worms transferred into fresh water, after spending a month in a sealed tube on Earth. Note that after being gathered and placed in the middle of a Petri dish, the worms immediately extend and begin to move and spread out, avoiding the bright light from above, similarly to normal worms.

Video S2. Worms after spending a month in a sealed tube in space, transferred into fresh water. Note that after being gathered and placed in the middle of a Petri dish, the worms will stay in a ventrally curled position as in a state of shock, and will not extend nor begin to move for an extended period of time, notwithstanding the presence of a bright light from above.
