# Supplementary figures and images for: Planarian regeneration in space: Persistent anatomical, behavioral, and bacteriological changes induced by space travel
Source: Regeneration (Oxf). 2017 Jun 13;4(2):85–102. doi: 10.1002/reg2.79 (PMC5469732; doi:10.1002/reg2.79)

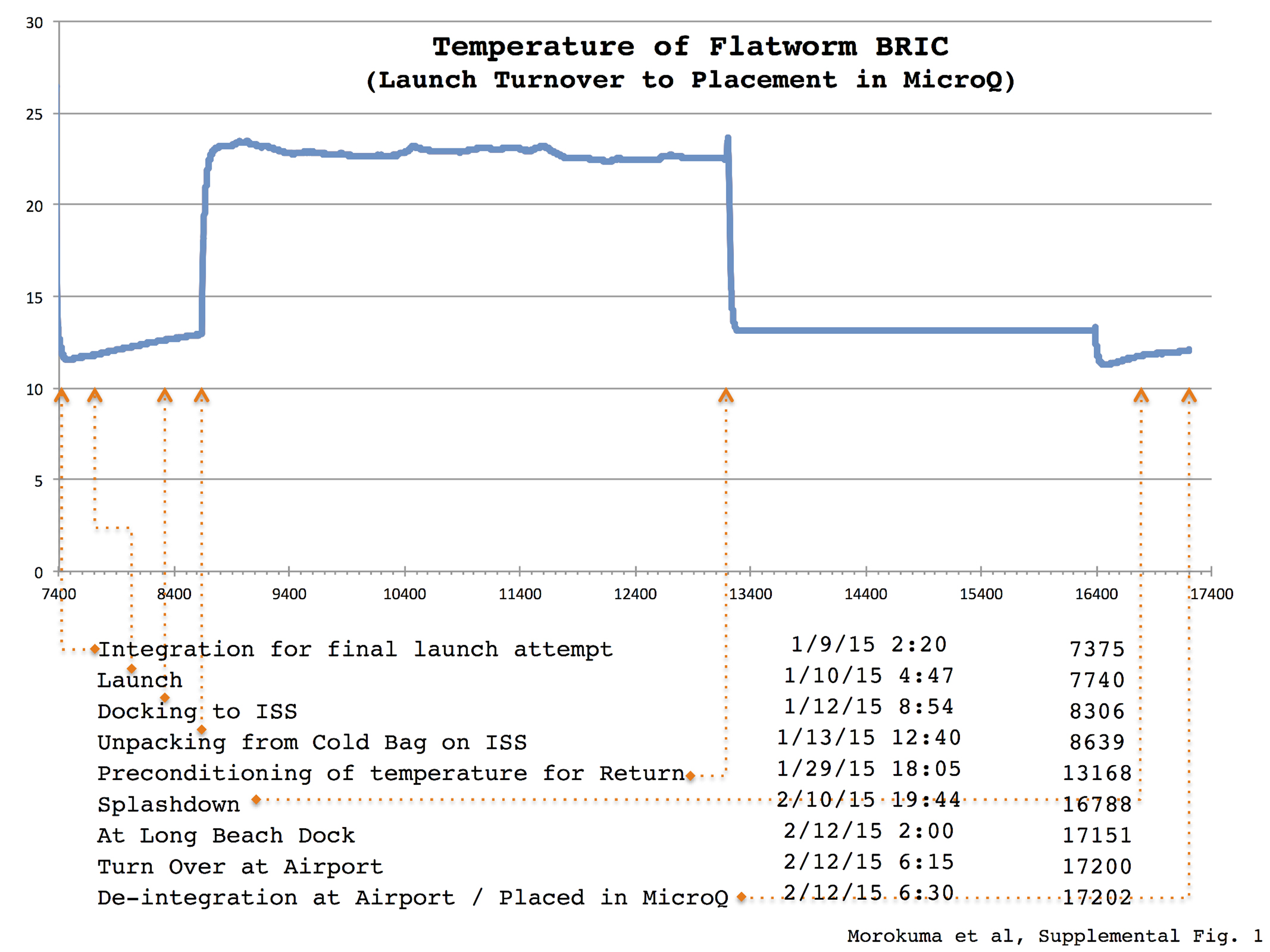

Supplement: Supplementary file 1 — Figure S1. Temperature profile for planaria during the SpX‐5 mission. The timing of critical events is also listed below the plot. [file REG2-4-85-s001.tif]

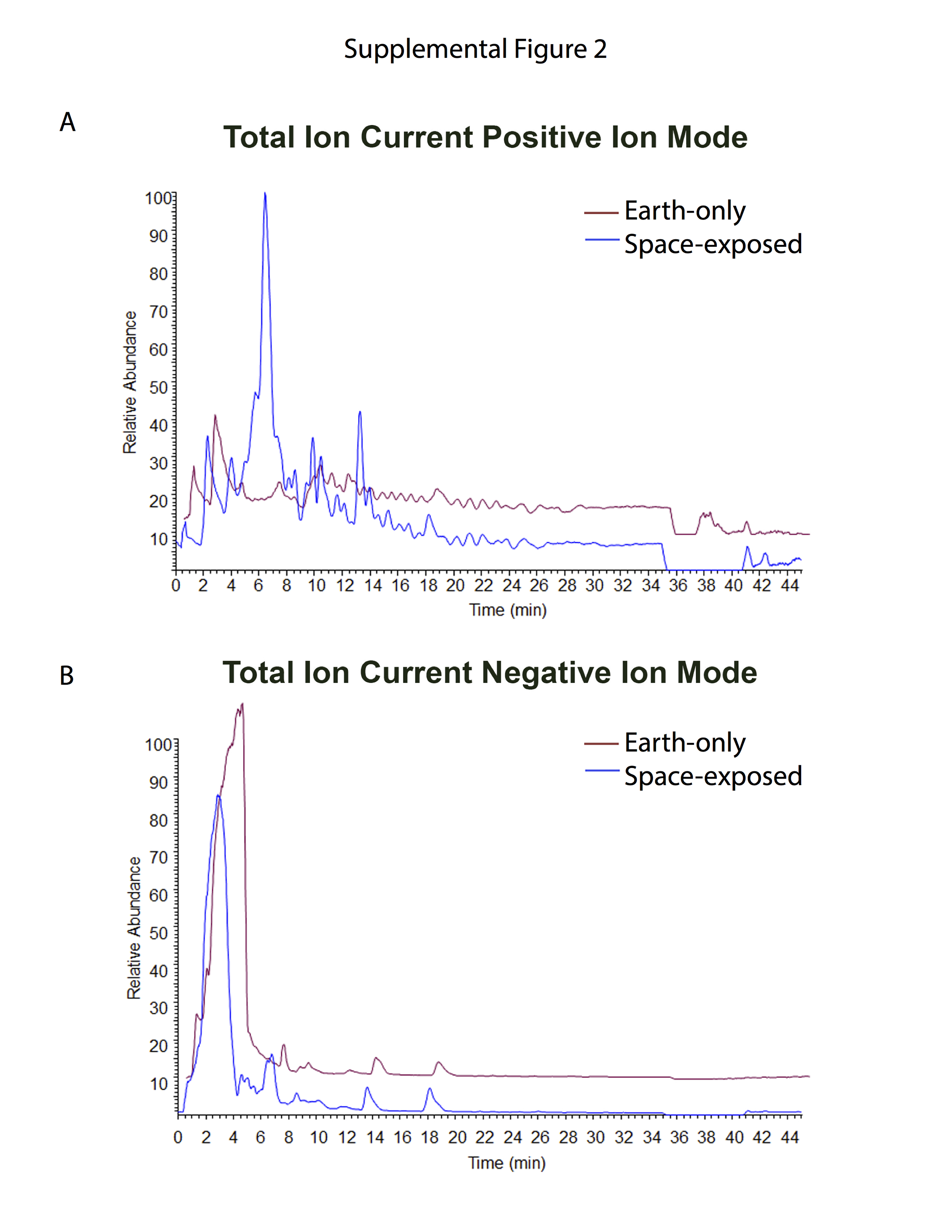

Supplement: Supplementary file 2 — Figure S2. Total ion chromatograms for Earth‐only (red) and space‐exposed (blue) worms. (A) The LC‐MS was run in positive ion mode. (B) The LC‐MS was run in negative ion mode. [file REG2-4-85-s002.tif]

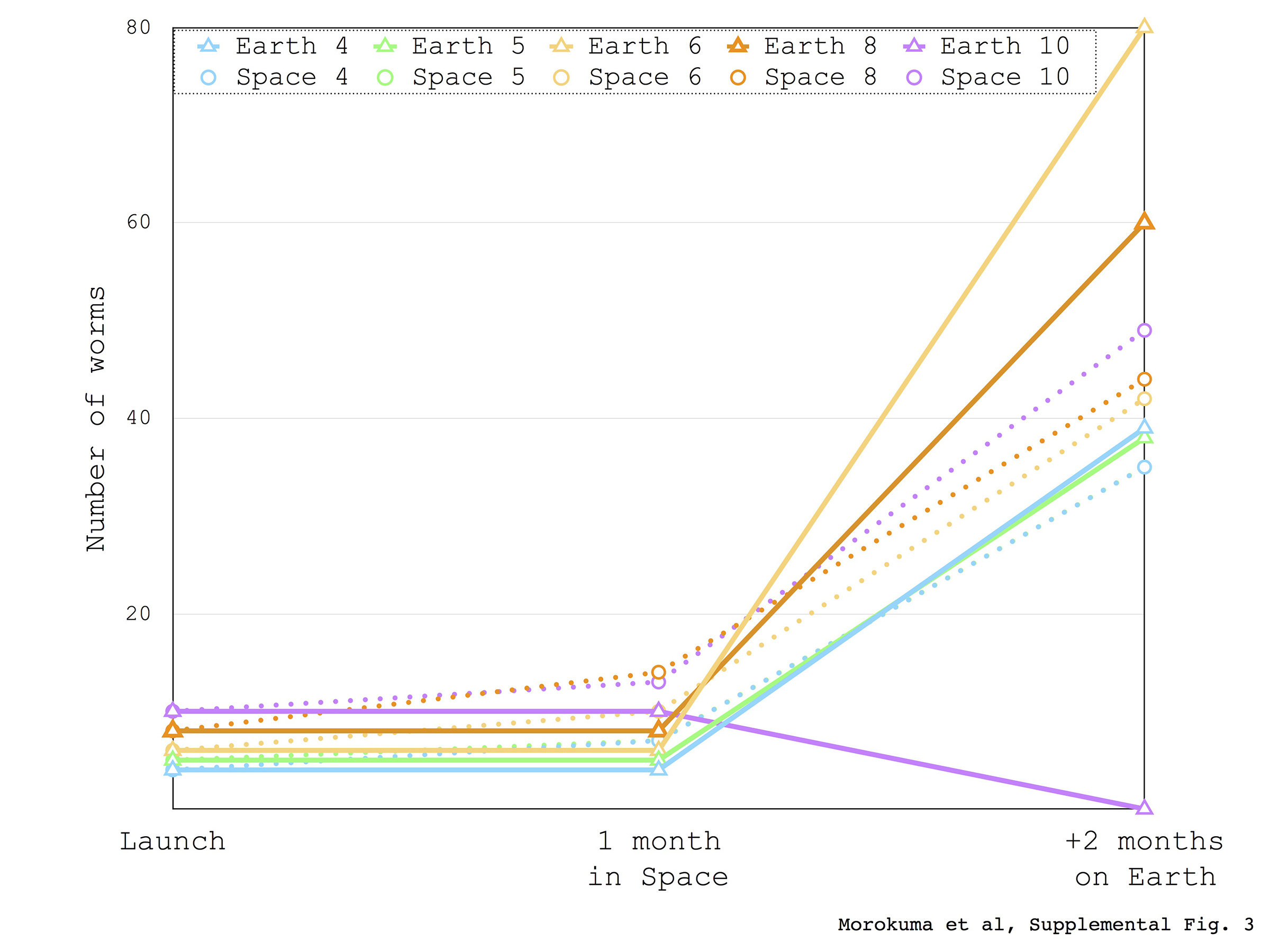

Supplement: Supplementary file 3 — Supplemental Figure 3. Worm colony growth before and after being in space. The number of worms before and after one month in a sealed tube, either while traveling to space and back (circles + dotted lines), or left on Earth (triangles + solid lines), together with number of worms after additional two months on Earth (same starting sample N = same color). Worm colonies which have traveled to space, all have shown slightly reduced rate in colony size growth, compared to their Earth counterparts. (See Supplemental Table 2.) Note that the colony from 10 whole worms left of Earth did not survive the duration of the additional two months on Earth for an unknown reason. [file REG2-4-85-s003.tif]
