# Supplementary material for: Planarian regeneration in space: Persistent anatomical, behavioral, and bacteriological changes induced by space travel
Source: Regeneration (Oxf). 2017 Jun 13;4(2):85–102. doi: 10.1002/reg2.79 (PMC5469732; doi:10.1002/reg2.79)
Supplement: Supplementary file 4 — Supplemental Data: Protein sequence alignments for the proteins identified in Supplemental Table 1 [file REG2-4-85-s004.docx]

**Supplemental Data: protein sequence alignments for the proteins identified in Supplemental Table 1**

**Pairwise blastp: comp145442_c0_seq1 vs. SMU15038343**

Score Expect Method Identities Positives Gaps

5687 bits (14753) 0.0 Compositional matrix adjust. 2719/3718(73%) 3193/3718(85%) 3/3718(0%)

Query 1 MITDLPVGITVINLKPNPSICTNTSMPTNEKSINLTYLAVNSDNLNNLTNVSSHCEQFVS 60

M TDLPVGITVINL+ NPS+C+N+++ + EKSINLTYLA N DN+NNLT+VSSHCEQFVS

Sbjct 2278 MKTDLPVGITVINLQSNPSLCSNSTLSSEEKSINLTYLAANQDNINNLTSVSSHCEQFVS 2337

Query 61 YDCQNSPITDGIKYGAYDNSGKYIEYWIGPKDKKFCANETCNCAVIDDKMRSDSGKFTDK 120

YDC+NSP+ DGIKYGAYD+S KY+EYW GP DK C+N++CNCAV+DDKMRSDSGKFTDK

Sbjct 2338 YDCRNSPLFDGIKYGAYDDSQKYVEYWTGPNDKNSCSNQSCNCAVVDDKMRSDSGKFTDK 2397

Query 121 TILPLSSISLPKVTGKRKLCINELRCYNLPKTCDEYKQQRRLDINKGNRNNIWAIDPDQA 180

LPLSSI +P G+RKLC+ ELRCYNLPKTCDEY Q +RLDINKGNRNNIWAIDPDQA

Sbjct 2398 FRLPLSSILMPSAPGQRKLCVKELRCYNLPKTCDEYNQLQRLDINKGNRNNIWAIDPDQA 2457

Query 181 GGETYFGVLCKMIDSVTVSETLQESNPLSINSNNNKATTVNIPFVNTSPLQIQNLVNQSN 240

GGE YFGVLCK ID V V+ET Q +NP ++NS+ K+T N+ F++TSP+QIQ LV+ SN

Sbjct 2458 GGEEYFGVLCKTIDGVVVTETRQTNNPQAVNSS--KSTVGNVSFIDTSPIQIQKLVSLSN 2515

Query 241 YCSQRVDYLCKNSGSLYNNSPKLFDFKNNPIITWAGADLYSAVGSCACDVLGSCPLNSKC 300

YCSQRVDY C NSG+LYN +PK+FD++N +++WAGAD Y AVGSCACDVLG+CP N KC

Sbjct 2516 YCSQRVDYFCTNSGTLYNKNPKMFDYQNKQLVSWAGADRYHAVGSCACDVLGNCPNNYKC 2575

Query 301 RCDALSNSTQYEGGIFTDRTILPIQRIQYQKGQSIQSNLYPVDCGSQPFDLPKDCIDART 360

RCDALSNSTQYEGGIFTD++ILP+Q++QYQ GQ+I +N+YPVDCGS FD+PKDCI+AR

Sbjct 2576 RCDALSNSTQYEGGIFTDQSILPVQQVQYQAGQNILTNMYPVDCGSSIFDIPKDCIEARN 2635

Query 361 KGYTYDTEILIKPNGVSKPYLVQCLMNGGKNKDLQITVVPVKVASIQNNESIPFIYPSAP 420

KG+ YDTE+LIKP+GV++P+LV C MN G NK+LQIT+V + N+S+ YP+

Sbjct 2636 KGFMYDTEVLIKPSGVTQPFLVFCQMNAGNNKNLQITMVLTNTTFSKTNQSVQITYPTTS 2695

Query 421 FNDIKQLVQNSLYCIQPMKANCKGILFSSVFTWTDGNNTTQTTFGTFNKNTFCPCGLNHV 480

ND KQLV+NS YCIQPMK NCKGI+FSS+FTWTDG+N Q FG+ N N FCPCGL +

Sbjct 2696 VNDAKQLVKNSAYCIQPMKMNCKGIMFSSIFTWTDGSNRNQLNFGSNNINDFCPCGLTNG 2755

Query 481 CAGIPGESKQSMFNRKCNCDTPNYNVSLSEYVLISTRNILPIKSLNFKLSPDSDPMNSVT 540

CAGI GESK SM +R+C+CDTP+ + + S+ VLI+ + +LPIKS KL P++DP+NS+

Sbjct 2756 CAGILGESKSSMMSRQCSCDTPDSSAAFSDSVLITNKTLLPIKSFILKLPPNADPVNSLV 2815

Query 541 VGSLMCANTRFDFDECATSFHDCDSHANCTNLDYNYRCDCNSGWQGLGGNEMYSNGRSCI 600

+GSLMC++++ DF+EC+T+FHDCD HANCTNLD +RCDC GWQGLGGNEMYSNGRSCI

Sbjct 2816 IGSLMCSSSKIDFNECSTNFHDCDLHANCTNLDSGFRCDCIKGWQGLGGNEMYSNGRSCI 2875

Query 601 DDNECALLKCPSVSDCLNTPGSFICTCHIGFQKTAPTVCIDIDECKRNSSICDQNARCVN 660

DD+ECALLKCPS SDC NTPGSFIC CH+GF K APTVC DI+EC NSSICD NA C+N

Sbjct 2876 DDDECALLKCPSTSDCTNTPGSFICNCHVGFVKAAPTVCNDINECSLNSSICDANADCIN 2935

Query 661 TYGSYLCTCNAGFRGSGKPGDCEAVATCGCWGDPHCLSFDGNWFHYQGRCKYTLVRDECE 720

TYGSY+C C GFRGSG PGDCEAVA CGCWGDPHCLSFDGNW HYQGRCKYTLVRDEC

Sbjct 2936 TYGSYICNCKPGFRGSGNPGDCEAVAICGCWGDPHCLSFDGNWLHYQGRCKYTLVRDECR 2995

Query 721 NGLPKDSSTPNFEVIMKNWDQNTGKNSMVSWAKEITVKIMNYTIMMKIGLFLIVNGQKTS 780

NGLP +SST NFEVIMKNWDQNTG NSMVSWAKEITVKIMNYTIMMKIG L+V+GQKTS

Sbjct 2996 NGLPIESSTANFEVIMKNWDQNTGTNSMVSWAKEITVKIMNYTIMMKIGFELVVDGQKTS 3055

Query 781 VPFNPKLNDGTNVGFEVAFFGSTLRLTCITGLEIKWDGVSRVDVTIPGRYMKNVCGQCGN 840

+PF PK DGT VGFEV+F+GS+LRLT + GLE+KWDG+S VDVTI YM NVCG CGN

Sbjct 3056 IPFIPKNEDGTPVGFEVSFYGSSLRLTSVHGLEVKWDGISMVDVTITSFYMNNVCGLCGN 3115

Query 841 YNLNPLDDWIVGPKCKPSGKTTDLLNLFGDSWRNDDPADTDPFCSSDTCNETPNEKPCDS 900

YN NP DDWIVGP CKPSG T+LLNLFGDSWRND+P DTDPFCS+ TC ETPNE PC +

Sbjct 3116 YNKNPHDDWIVGPNCKPSGNITELLNLFGDSWRNDNPIDTDPFCSTTTCKETPNETPCAA 3175

Query 901 ALMTKSKLECEKLREKFLACEIVMRQMNHSLDEYIESCVYDQCYSTGDLTQMMCKTAESL 960

+M S+LEC+KLR+KF +CE VM+ N SLDEYIESCVYDQCYS+GDLTQMMCKTAESL

Sbjct 3176 DVMAHSQLECQKLRDKFASCETVMKTFNKSLDEYIESCVYDQCYSSGDLTQMMCKTAESL 3235

Query 961 AQKCLEEYKVKVLYRSVNFCNMVCNKNMVYSSCASPCQPTCYNNTNSMLCNGPCVESCVC 1020

AQKCLEEYKVK+ YRS+NFCNMVCNKNM+YS CASPCQPTCYNNTNSMLC G CVESC+C

Sbjct 3236 AQKCLEEYKVKISYRSINFCNMVCNKNMIYSDCASPCQPTCYNNTNSMLCTGQCVESCIC 3295

Query 1021 VDGYVMENGICINPKSCGCLMSDGTYYSNGEQRTNEICSMKCGCNGKTGQLECSNITCSN 1080

GYV+ENG C P++CGCLMSDGTYY+NGEQRTNE CS+KC C G+TGQLEC+NITCSN

Sbjct 3296 APGYVLENGNCTKPEACGCLMSDGTYYANGEQRTNENCSVKCRCKGETGQLECTNITCSN 3355

Query 1081 DAFCDFKDNDYGCHCKNGFMGDGIICKDIDECSLIPSVCDINADCKNSIGSYSCSCKEGF 1140

DAFCDFKD+DYGCHCKNGFMGDGI+CKDIDECS SVCD NA CKN+IGS+SCSCKEGF

Sbjct 3356 DAFCDFKDDDYGCHCKNGFMGDGIMCKDIDECSNNTSVCDSNAFCKNTIGSFSCSCKEGF 3415

Query 1141 EGNGKTCNNINECYPLSPCDNSTEECFDQIPGYQCRCKNGFLKNMTTGKCEDKNECADTG 1200

EGNG TC NINECYPLSPCDN+TEECFD++PGY+CRC+ GF+KN TTG C+D+NECADTG

Sbjct 3416 EGNGVTCTNINECYPLSPCDNATEECFDKVPGYECRCQKGFMKNTTTGNCDDRNECADTG 3475

Query 1201 NLCDRVSTNCNNTFGSYRCDCKYGFRPSPIDSFVCTDVNECNLVHECDKNYAKCTNTPGS 1260

NLCDRVSTNCNNTFGSYRCDCKYGFRPSPIDSFVCTDVNECNLVHECDKNYAKCTNTPGS

Sbjct 3476 NLCDRVSTNCNNTFGSYRCDCKYGFRPSPIDSFVCTDVNECNLVHECDKNYAKCTNTPGS 3535

Query 1261 YYCTCDSGFQGDGRNCTDIDECITKKVCQRPDATCVNLPGSYECRCLNGKPGCDGDNPCN 1320

YYCTCDSG+QGDGRNCTDIDEC + KVCQRPDATCVNLPGSYECRCL+G PGCDGDNPCN

Sbjct 3536 YYCTCDSGYQGDGRNCTDIDECSSNKVCQRPDATCVNLPGSYECRCLDGTPGCDGDNPCN 3595

Query 1321 TVRCNHLNEICYLGQCFCKRGFERNITTNQCEDINECETAANDCAERIAKCVNLDGSYEC 1380

TV+C NEICYLGQCFCK GF+ N+TTN CEDI+EC+TAANDCA R AKC NLDGSYEC

Sbjct 3596 TVQCTKPNEICYLGQCFCKHGFQNNVTTNICEDIDECDTAANDCAGRKAKCANLDGSYEC 3655

Query 1381 ECLYGYRMSPVNNSCENINECAEDLHKCKENAKCVDTKGGYYCECQTGFTGHCDECRDID 1440

+CLYGYRMS +N CENINEC ++LH+C ENA+C+DT GGYYC+C+TGFTGHCDECRDID

Sbjct 3656 DCLYGYRMSSINKICENINECIDNLHQCGENAECIDTDGGYYCQCKTGFTGHCDECRDID 3715

Query 1441 ECVLSLHKCDQDYGTCENSIGSYVCRCNEGYSGDGSICSDINECLIGVHNCSNLNQYCLN 1500

EC SL+ CD D TCENSIGS+ CRCNEGYSGDGS+CS+I+ECL+G+HNCS +Q+C N

Sbjct 3716 ECAFSLNNCDSDRATCENSIGSFTCRCNEGYSGDGSVCSEIDECLLGLHNCSRSHQFCFN 3775

Query 1501 VDGGYECKCLSGYKMDIDGTCSDINECSFSDNGCDDVAHCENIQGSFRCNCPNGYTGNGK 1560

V+GG+EC+CLSGYK D +G+C D NEC F DNGCDD A C N GSF C C GY G+G+

Sbjct 3776 VNGGFECRCLSGYKSDTNGSCVDENECRFFDNGCDDNADCINTDGSFLCICRAGYIGSGR 3835

Query 1561 TCTPIDGHEKCGRLLCPLNAICENSTCQCKHGYKNSSNSCENINECKNQTSCDVNANCID 1620

C P +G E+CG+LLCPLNA+C NSTC CK GY+NS+N C N +EC++QTSCDVNANCID

Sbjct 3836 NCDPQEGLEECGKLLCPLNALCVNSTCLCKSGYENSTNQCINTDECESQTSCDVNANCID 3895

Query 1621 THGSFICYCNEGYVGDGYNCYKKTTEIDICDTDSFCIDGDCYDGECHCPQGYVFKANKCI 1680

THGSFICYCNEGY+GDG NC+KK T+ D C +++C DGDCYDG CHCP G+VFK+NKC

Sbjct 3896 THGSFICYCNEGYIGDGINCFKKDTDTDACQIENYCTDGDCYDGICHCPSGFVFKSNKCF 3955

Query 1681 DERCKNVCPKTAICSISSGSPVCSCSVGTSLSSDNSECIDIDECRDNSHDCQMNSFCLNR 1740

DERC+NVCPK AICSISSGSPVCSCSVGTSLSSD+S+CIDIDEC +N DCQ NS C NR

Sbjct 3956 DERCENVCPKNAICSISSGSPVCSCSVGTSLSSDSSQCIDIDECNENVDDCQENSICFNR 4015

Query 1741 FGSYDCKCPKNNVDVFNDGKICIAVQPAKCDHECPIGQYCNSGNCSCLPGLNAKLDNE-H 1799

FGSYDCKCP N +DVF DGK+C+AV ++C+ CP GQYCN+G+CSCL GLN++ D++ +

Sbjct 4016 FGSYDCKCPPNYLDVFKDGKVCMAVPLSQCNQSCPTGQYCNNGSCSCLMGLNSQTDDDGN 4075

Query 1800 LICNKISDSCLPGTCPLNADCEERLFGYLCKCKAGYEGVGVKSCRDIDECGSKTDNCTIN 1859

L C+K S SCLPG CPLNA+CEERLFGY+CKCK GYEGVG+KSCRDIDECG K +NCTIN

Sbjct 4076 LNCSKNSASCLPGICPLNANCEERLFGYICKCKPGYEGVGIKSCRDIDECGGKINNCTIN 4135

Query 1860 ENCNNIPGSFNCLCKKGYSRNSTTGFCETNNKCNCGPHGICGNDSKCLCKPGYQVNEKGV 1919

E+C+N GSF C CK+GYSRNS++G CETNN CNCG HGICG + C CK GY++N G+

Sbjct 4136 EDCSNTEGSFTCSCKRGYSRNSSSGLCETNNMCNCGSHGICGPNYICQCKSGYEINPSGM 4195

Query 1920 CEDVDECQTQSPCHMLAQCTNTLGSYKCSCPENFYGDPENKCIENKCKTKALQCQIGEEC 1979

C+DV+ECQTQSPCH+LAQC NT GSYKC CPENFYGDP+NKC E+KCKTKALQCQ GE C

Sbjct 4196 CQDVNECQTQSPCHILAQCINTPGSYKCQCPENFYGDPQNKCFEDKCKTKALQCQAGEVC 4255

Query 1980 KLSLYGSVCQKKICNSTEILVDNECLPVNTLCQAVSCGTNAVCKIENGRPDCFCDSGYSG 2039

KLSLYGSVC+K CNS+EIL++NECLPVN +CQ ++CG +A CKIENGR DCFCDSGY G

Sbjct 4256 KLSLYGSVCEKISCNSSEILINNECLPVNQVCQNINCGNHAFCKIENGRADCFCDSGYYG 4315

Query 2040 DGEKCSDIDECSNGLVNCPNNSFCQNKDGSYHCACNIGFQRPGNSTASDPCTDIDECVIP 2099

DGE C D+DECSNG V+CPNNSFCQNKDGS+HCACN+GFQR N+ +D CTDIDEC++

Sbjct 4316 DGELCVDVDECSNGDVSCPNNSFCQNKDGSFHCACNVGFQRLENTLVTDSCTDIDECLLS 4375

Query 2100 QSCATNALCENSIGSFRCECKEGYIGDGKYSCQIDSKCKRHGGCHEKAACILIAEKSSYT 2159

+SCATN C+N+IGSF+CECK+G+IGDGKY C+IDSKC+++GGCHE A+CI+ E S Y

Sbjct 4376 ESCATNGECQNTIGSFKCECKDGFIGDGKYDCRIDSKCEKYGGCHEDASCIVNVESSIYE 4435

Query 2160 CQCNSGYYGDGINNCIKNNLCFENGQSKCHENAICQQLNATYQCICPSGFLGDGYNFCND 2219

CQC++ + GDGI +CIKNNLCF NG+S CHENAIC+Q+NATY+CICP GF GDGYNFCND

Sbjct 4436 CQCHTEFSGDGITSCIKNNLCFVNGKSSCHENAICEQVNATYRCICPDGFQGDGYNFCND 4495

Query 2220 INECSNKNSHNCSKLEKCVNLDGDYKCVCVDGADIVNNVCVDIDECSSNTTNKCSSDAVC 2279

I+EC+N N+HNCS LEKCVN +G Y CVC DGA V+++CVDIDECSSN TNKCSS+A+C

Sbjct 4496 IDECANDNTHNCSSLEKCVNSNGQYNCVCGDGAVYVDDICVDIDECSSNQTNKCSSNAIC 4555

Query 2280 KNKIGTYTCQCKDGFFGNGILCHDIDECKRGLANCSENALCINKPGSFACECQAGTIGNG 2339

+NK GTY CQC +GF+G+G+LCHDIDECK G+ANCSENALCINKPG+FACECQAGT+GNG

Sbjct 4556 QNKEGTYGCQCAEGFYGDGLLCHDIDECKFGVANCSENALCINKPGTFACECQAGTLGNG 4615

Query 2340 TTCEDEDECKKPKGTSGAAECDENAVCKNKDPGYTCVCNSGYKGTGWFCVKETPCDKPNA 2399

TTC DEDECKKPKG+ GA ECDENAVCKNKDPGYTC C SGYKGTGWFC+K+TPCD PNA

Sbjct 4616 TTCGDEDECKKPKGSPGAPECDENAVCKNKDPGYTCECQSGYKGTGWFCIKQTPCDVPNA 4675

Query 2400 CSSNQTCTPSDDNSLAICECKPEFKLENNNCVSKQECDNDIDTCDRQTSNCIQLDRGFKC 2459

C +NQTCTPS++NSLAICECKP+FKLEN+ C+ K EC+N D CDR+TSNC+QLD GFKC

Sbjct 4676 CKANQTCTPSNENSLAICECKPDFKLENDTCIPKTECENKTDNCDRETSNCVQLDPGFKC 4735

Query 2460 ECKPGFKMVGNTCQDKNECDPTSTDFAAGECFKTGGGCINTPGGFECTCSAKQETQENNS 2519

ECKPGF MVGNTCQDKNECDPTS DFAA +C +GGGC+NT G F CTC+ +Q + N+S

Sbjct 4736 ECKPGFNMVGNTCQDKNECDPTSPDFAAQKCLASGGGCLNTKGSFVCTCNQEQMNENNSS 4795

Query 2520 CKPIDSCALKLDKCDKKVENCISNKDSSYTCKCIEGFEKVDNHCVDIDECANKIDSCKNN 2579

CKPIDSCA+ LD CD+KVE+CIS+KDSSYTC+CIEGF++++ CVD+DECA+KIDSCKNN

Sbjct 4796 CKPIDSCAMGLDNCDRKVEDCISSKDSSYTCQCIEGFQRINGSCVDVDECADKIDSCKNN 4855

Query 2580 ATCTNTIGSYTCNCPENLKLDTSKKSCTDRNECKENPMICGELSKCLNTDGSYKCVCVTG 2639

ATC N IGSY C CP+NLKLD SKK+CTDRNEC+ENPMICG+LSKCLNTDGSY+C CVTG

Sbjct 4856 ATCNNMIGSYVCKCPDNLKLDLSKKACTDRNECQENPMICGDLSKCLNTDGSYECQCVTG 4915

Query 2640 YKWNGTHCEDIDECANKTHPCHEFAQCSNVPGSCKCKCKQGYTGDGIYSCQEIDKCPSRE 2699

YKWNGT+CEDIDECA KTHPCH+ A+CSNVPGSCKCKC+ G+TGDGIYSC E D CP+R+

Sbjct 4916 YKWNGTNCEDIDECAIKTHPCHDLARCSNVPGSCKCKCQPGFTGDGIYSCIETDSCPARD 4975

Query 2700 DVKCPVGTYCNTIGNIVYCNCTSGYEDSQNMECIGDVCYKDCNDINECDTKKSACHELAK 2759

DVKCP G+YCN IGN +YCNCT+GYEDS MECIGDVCYK C+DI+EC T+KSACH+ A+

Sbjct 4976 DVKCPTGSYCNMIGNFIYCNCTNGYEDSNQMECIGDVCYKKCSDIDECVTRKSACHKFAE 5035

Query 2760 CTNNKGGYVCTCPSYLVGDGKISCKDQNECALEIHSCDVNTSYCENLDNSMDVLEPYQCH 2819

CTNN G YVCTCPSYL GDGK +C+DQNECALE H+C++NTSYCENLDN+++VL P+QCH

Sbjct 5036 CTNNDGAYVCTCPSYLTGDGKTTCEDQNECALETHTCNLNTSYCENLDNTVNVLAPFQCH 5095

Query 2820 CFNGYVKIPGTQICVNKNECLNPAENDCVENSVCVDTVGSFECKCENGFKQLPTGKCVDI 2879

CFNGYVKIPGTQIC+NKNECLNP ENDCVEN++C+DT GS+EC+C++GFKQLP+GKC +I

Sbjct 5096 CFNGYVKIPGTQICLNKNECLNPVENDCVENAICIDTEGSYECRCKSGFKQLPSGKCENI 5155

Query 2880 DECEEKLDNCSRNSVCVNRIGSYYCECNSGFHWSDNTYINCEDINECDNPTSCNLLSNSL 2939

+ECEEK DNCS NSVC ++ + C+C GF WSD T +NCEDI+EC NPTSC+ + +S+

Sbjct 5156 NECEEKSDNCSINSVCQDKADGFSCQCKPGFKWSDETNLNCEDIDECTNPTSCSSMPHSI 5215

Query 2940 CVNNPGSFFCLCQPGMSLQGGQCTDINECLKENSCGKNAKCTNTVSSYNCSCSDGLRKLP 2999

C+NNPGSF C+C G SLQGG C DINECL NSCG+N+ C NTVS +NCSC++G R+LP

Sbjct 5216 CMNNPGSFECVCPHGFSLQGGYCNDINECLIPNSCGQNSVCANTVSGFNCSCANGARQLP 5275

Query 3000 NGDCELINECLEGIDRCEELNGANSTCYDLPVGYGCVCPKGFVNDRKLAQICENQNECEI 3059

NGDCELINECLE IDRC+ LNGA+S CYDLP+GY CVCP GFVND Q+CENQNECEI

Sbjct 5276 NGDCELINECLENIDRCQVLNGADSVCYDLPIGYACVCPDGFVNDEVFPQVCENQNECEI 5335

Query 3060 NIHNCSVANSDCVDTYGSFKCECKSGYSQNSQGDCVDIDECKISEKNQIDLCNTGKCVNT 3119

NIH+C +NS+CVDTYGSFKC CK G+S+NS GDCVD DEC+ISE++ + CN GKC+NT

Sbjct 5336 NIHSCQASNSECVDTYGSFKCVCKPGFSKNSDGDCVDYDECEISEQSNMKFCNNGKCINT 5395

Query 3120 KGSYICECPSGFVNDNNNCTDVNECLLYNGTNESNNCDHENGYCINSEGSFACRCKEGYS 3179

G + C+CP GF+ +N +C DV+EC L ++ ++NCD NG+CIN+ G F C C G+

Sbjct 5396 PGGFTCDCPEGFIYNNYDCIDVDECNLDQVSSLADNCDQVNGHCINTVGGFQCYCNRGFV 5455

Query 3180 MLENSTVCQDIDECNFTNSCLDGLCNNLPGSFECLCKKGYNLVNGKCEDVDECFNDSLKC 3239

M+ENST C DIDEC F NSCL+GLCNNLPGSFECLC GY+LVNGKC D++EC N+S C

Sbjct 5456 MIENSTKCVDIDECQFPNSCLNGLCNNLPGSFECLCNVGYDLVNGKCTDINECSNNSQIC 5515

Query 3240 GPEIAGVCLNKIGSYQCKCNVGYENSGGKSYNPCQQINECVKWNVTCPANAQCVDRSVGY 3299

GP+ AG+C+N +GS+QCKCNVGYENSGGKSYNPCQ +NECVK N+TCP+NAQCVDRS GY

Sbjct 5516 GPQAAGICINAVGSFQCKCNVGYENSGGKSYNPCQSVNECVKLNITCPSNAQCVDRSNGY 5575

Query 3300 ECICKDGYQEDQIGVCRNINECKIDNTCHYRAKCVDTDGSYKCICKYGYKGDGKVSCKAI 3359

ECICKDGYQEDQIG+CRNINEC+I+++CHY A C+DTDGSYKC CKYGYKGDG VSCKAI

Sbjct 5576 ECICKDGYQEDQIGICRNINECQIEDSCHYLADCLDTDGSYKCTCKYGYKGDGIVSCKAI 5635

Query 3360 CGPNSCPAGQLCKIINGDQFECSCSCEGERCRETGPVCNTRGMTYSSEKEMFESSCKLNI 3419

CGPNSC GQLC+I+NG+Q++C C C+G RCR+ GPVC+T+G+TY SEK+MFE++CK NI

Sbjct 5636 CGPNSCLPGQLCQIVNGNQYDCGCVCQGPRCRDNGPVCDTQGITYQSEKQMFETTCKQNI 5695

Query 3420 STEVEYYHACQQSCSKVVCPGNEKCTISKGKPICSCEECTAADRTPRVLCGSNGIEFKSV 3479

EVEYY+ CQ+SC+ V CPG EKC++ +P C+C+ CT A+ TPR+ C +NGIEF S+

Sbjct 5696 VGEVEYYNECQKSCATVTCPGIEKCSMVNDRPSCTCQNCTQAELTPRLFCSNNGIEFHSI 5755

Query 3480 CQMKTWMCNSKTEISISYDGPCHKSIDCSVSEWTNWSSCSKTCGIGRSSRTRMVLKVALF 3539

CQMKTW+CN+K+EISISYDG CH+SIDC VSEWT+WSSCSKTCG+GR +RTR+++K A+F

Sbjct 5756 CQMKTWICNTKSEISISYDGSCHRSIDCEVSEWTSWSSCSKTCGVGRYTRTRIIVKAAMF 5815

Query 3540 NGECDEVLSETQQCYNGPCPGDECENISCPPSSFCEFGKCVCPDCSNQKTSDPVCAKIGE 3599

NG C++ L ETQQCYNGPCPGD CENI+C P SFCE GKC+CPDCS+Q+ DPVC KIG+

Sbjct 5816 NGNCEDPLFETQQCYNGPCPGDACENITCAPGSFCESGKCICPDCSDQRIPDPVCGKIGD 5875

Query 3600 SQSGTFRSFCTLLHNACMLNSTFTYLHKGICGDHVPSEPKICTMVTNFKIVQSNDNCTST 3659

+SGT+R+FCTLLHNAC NS+FTYLH+G CG+H+PSEPKIC+MVT+F+IVQS DNCTS

Sbjct 5876 LESGTYRTFCTLLHNACNYNSSFTYLHRGRCGEHIPSEPKICSMVTHFQIVQSQDNCTSV 5935

Query 3660 EPLRVNLCSGGCGKNPKYCCRPENNRILRARFICPDNSYVIRQVKSISSCECKLEENI 3717

EP+RVNLCSGGCGKNP YCCRP RI R++F CPDNS+V R+VKSISSCECKLEEN+

Sbjct 5936 EPVRVNLCSGGCGKNPNYCCRPAEERIFRSKFRCPDNSFVYREVKSISSCECKLEENL 5993

**Pairwise blastp: comp128998_c0_seq1 vs. SMU15000643**

Score Expect Method Identities Positives Gaps

789 bits (2038) 0.0 Compositional matrix adjust. 372/453(82%) 405/453(89%) 0/453(0%)

Query 1 MILAILLSFCLVQLSVSDTPANCTYQDVIGKWTVYTGNFSVSCSTSQFVATKTLTLIYPN 60

MIL I+LS L+ ++SDTPANC+YQD IGKW V+T NFS SCSTS FV TKTLTLIYPN

Sbjct 1 MILRIILSIGLLHFALSDTPANCSYQDAIGKWQVFTSNFSTSCSTSAFVPTKTLTLIYPN 60

Query 61 LAIDEFGNYGKWTLIYNQGFEVIINNKKYFGFFDYKKINSTYAISYCDRLQPNWFHDVLI 120

LAID+FGN+G WTLIYNQGFEV I NKKYFG+FD+K+INSTY ISYC+RLQPNW+HDVLI

Sbjct 61 LAIDDFGNFGTWTLIYNQGFEVTITNKKYFGYFDFKQINSTYTISYCNRLQPNWYHDVLI 120

Query 121 RQWQCFKAQKVSSLKEKNNLSPNSNVFALTSLRFGSQKRIVDKINMGNNGWTAKDYPEFH 180

RQW+CFKAQK+S+L EK+ P SN+FAL SL +GSQK IV+ IN N GW AKDYPEFH

Sbjct 121 RQWRCFKAQKLSNLGEKHVHIPQSNIFALNSLLYGSQKHIVNTINNQNIGWIAKDYPEFH 180

Query 181 EKTLYEITNMAGGSRSKLQRPKPAPITKSILESVKLIPKSFDWRNVNGVNYVSPVRNQGG 240

EKTLY+I MAGG SKL RPKPAP+T+ L SVK IPKSFDWRNVNGVNYVSPVRNQGG

Sbjct 181 EKTLYQIVRMAGGFSSKLHRPKPAPVTEYHLNSVKYIPKSFDWRNVNGVNYVSPVRNQGG 240

Query 241 CGSCYSFASAGMLEARYRIKSNNTILPILSPQDVVECSPYSQGCDGGFPYLIAGKFAEDF 300

CGSCYSFASAGMLEARYRIKSNNTI PILSPQDVVECSPYSQGC+GGFPYLIAGKFAEDF

Sbjct 241 CGSCYSFASAGMLEARYRIKSNNTISPILSPQDVVECSPYSQGCEGGFPYLIAGKFAEDF 300

Query 301 GMAQENCNPYKGVDGKCSTTKNCKRYFATNYKYIGGYYGATNEPLMRMELVRNGPIAVGF 360

GMAQE+CNPYKG DGKCSTTKNC+RY+AT YKYIGG+YGATNEPLMRMELVRNGPIAVGF

Sbjct 301 GMAQESCNPYKGEDGKCSTTKNCQRYYATKYKYIGGFYGATNEPLMRMELVRNGPIAVGF 360

Query 361 EVYNDFMGYRGGVYHHNFATRILTTSKFGFNPFELTNHAVLVVGYGETQSGEKFWTVKNS 420

EVY+DFM Y+ GVYHH+F TR LT SKFGFNPFELTNHAVLVVGYGET +GEKFWTVKNS

Sbjct 361 EVYDDFMSYQSGVYHHDFKTRKLTASKFGFNPFELTNHAVLVVGYGETATGEKFWTVKNS 420

Query 421 WGVGWGESGYFRIRRGNDECGIESLGVASEPIL 453

WG GWGE+GYFRIRR NDECGIESLGVASEPIL

Sbjct 421 WGSGWGENGYFRIRRANDECGIESLGVASEPIL 453

**Pairwise blastp: comp127150_c0_seq1 vs. SMU15003136**

Score Expect Method Identities Positives Gaps

3455 bits( 8960) 0.0 Compositional matrix adjust. 1714/1973(87%) 1864/1973(94%) 1/1973(0%)

Query 1 MVTIEAKNLQYLQVDKSLVSDSANLSDWANKKLVWIPDEIEGFISGSLIEEKGDEATVKL 60

M I+AK+LQYLQVDKSLVSDSANLSDWANKKLVWIPD+ EGFISGSLIEEKGDEATVKL

Sbjct 1 MENIDAKDLQYLQVDKSLVSDSANLSDWANKKLVWIPDDNEGFISGSLIEEKGDEATVKL 60

Query 61 ENGKSVKFPIENIQKMNPPKFLKSEDMADLTYLNEASVLFNLKDRYFSDLIYTYSGLFCV 120

ENGK++K PIEN+QK+NPPKF KSEDMADLTYLNEASVL+NLKDRYFSDLIYTYSGLFCV

Sbjct 61 ENGKTMKLPIENVQKVNPPKFFKSEDMADLTYLNEASVLYNLKDRYFSDLIYTYSGLFCV 120

Query 121 VVNPYKRLPIYTDNVIEWYKGRKRHERPPHIYAVTDVAYRNMLQDKENQSILCTGESGAG 180

VVNPYKRLPIYT+NVIEWYKGRKRHERPPHIYAVTDVAYRNMLQDKENQSILCTGESGAG

Sbjct 121 VVNPYKRLPIYTNNVIEWYKGRKRHERPPHIYAVTDVAYRNMLQDKENQSILCTGESGAG 180

Query 181 KTENTKKVIQYLASVATSLKNQKTTANNALSQFYAHDVNIGELETQLLQANPILEAFGNA 240

KTENTKKVIQYLASVATSLKNQKTTA+NALSQFYAHDVNIGELETQLLQANP+LEAFGNA

Sbjct 181 KTENTKKVIQYLASVATSLKNQKTTASNALSQFYAHDVNIGELETQLLQANPVLEAFGNA 240

Query 241 KTIKNDNSSRFGKFIRINFDNSGFISSANIETYLLEKARVIRQAASERCFHIFYQLLIGA 300

KTIKNDNSSRFGKFIRINFD SGFISSANIETYLLEKARVIRQAA+ERCFH+FYQLL+GA

Sbjct 241 KTIKNDNSSRFGKFIRINFDTSGFISSANIETYLLEKARVIRQAANERCFHVFYQLLLGA 300

Query 301 DDNLKKELILENVSTYKLLSNGMITVPDYDERQMFKDTVESLDIIGISKDEQDSIFRVIS 360

DD LKKELILENVSTYKLLSNGMI VPDYDE+QMFKDTVE+LDI+GISKDEQ+SIFRVI+

Sbjct 301 DDRLKKELILENVSTYKLLSNGMIAVPDYDEKQMFKDTVEALDIMGISKDEQESIFRVIA 360

Query 361 AVLHMGNIEFKQERSSDQAALPDNTVAQKVAHLLGLPVTEMTKALLKPKLKVGREVVAKA 420

AVLHMGNIEFKQERSSDQAALPDNTVAQKVAHLLGLPVTEMTKALLKPKLK+GREVVAKA

Sbjct 361 AVLHMGNIEFKQERSSDQAALPDNTVAQKVAHLLGLPVTEMTKALLKPKLKIGREVVAKA 420

Query 421 QTKEQAEFSVEAISKSTYERMFRWLVMRINRSIDRNRQKTNFIGILDIAGFEIFEINSFE 480

QTKEQAEFSVEAISKSTYERMFRWLVMRINRSIDRNRQKTNFIGILDIAGFEIFEINSFE

Sbjct 421 QTKEQAEFSVEAISKSTYERMFRWLVMRINRSIDRNRQKTNFIGILDIAGFEIFEINSFE 480

Query 481 QLCINYTNEKLQQLFNHTMFILEQEEYRKENIKWEFIDFGLDLQPTIDLIEKPMGILSLL 540

QLCINYTNEKLQQLFNHTMF+LEQEEYRKENIKWEFIDFGLDLQPTI+LIEKPMGILSLL

Sbjct 481 QLCINYTNEKLQQLFNHTMFVLEQEEYRKENIKWEFIDFGLDLQPTIELIEKPMGILSLL 540

Query 541 DEECFFPKATSKTFVEKIIKNQSSHSKFKATDFRAKADFGVIHYAGKVDYVADNWLVKNM 600

DEECFFPKAT+KTFVEK+IKNQS+H K KATDFRAKADFGVIHYAGKVDYV+DNWLVKNM

Sbjct 541 DEECFFPKATTKTFVEKLIKNQSTHPKLKATDFRAKADFGVIHYAGKVDYVSDNWLVKNM 600

Query 601 DPLNENVVSLLQESNELFVQTIWKDTENIIGLSTTTAQESAFGGAKT-RKGMFRTVGQLY 659

DPLNENVVSLLQESNE FVQTIWKD ENIIGLSTTTAQESAFGGAKT RKGMFRTVGQLY

Sbjct 601 DPLNENVVSLLQESNESFVQTIWKDAENIIGLSTTTAQESAFGGAKTTRKGMFRTVGQLY 660

Query 660 KESLTKLMDVLNNTSPNFVRCIIPNHEKKAGKIDSRLVIDQLKCNGVLEGIRICRQGFPN 719

KESLTKLMDVLNNT+PNFVRCIIPNHEKK+GKIDS+LVIDQLKCNGVLEGIRICRQGFPN

Sbjct 661 KESLTKLMDVLNNTNPNFVRCIIPNHEKKSGKIDSKLVIDQLKCNGVLEGIRICRQGFPN 720

Query 720 RILFQEFKQRYGILTPNVIPKGFMDGRKATELMLGALDIDVSNYRIGQSKIFFKAGVLAR 779

RILFQEFKQRYGILTPNVI KGFMDGRKATELMLG LDID SNYRIGQSKIFFKAGVLAR

Sbjct 721 RILFQEFKQRYGILTPNVILKGFMDGRKATELMLGELDIDASNYRIGQSKIFFKAGVLAR 780

Query 780 LEEDRDTKLTEIIVKFQSFARGYLARKNLQNRSQHLNAVKIIQRNCTAYLKLRNWSWWKL 839

LEEDRD KLTEIIVKFQS+ARGYLARKNLQNRSQ+LNA+KIIQRNC AYLKLRNW+WWKL

Sbjct 781 LEEDRDIKLTEIIVKFQSYARGYLARKNLQNRSQNLNAIKIIQRNCNAYLKLRNWAWWKL 840

Query 840 FTRVKPLLSVTRQEEVVALKEEELKKTKESLEKMTNEFEVTKKNYESLTEEKSKLQEDLE 899

FTRVKPLLSVTRQEEVVALKEEELKK+KE+LEKMT +FE TKKNYE L EEK+K+QEDLE

Sbjct 841 FTRVKPLLSVTRQEEVVALKEEELKKSKETLEKMTADFETTKKNYEFLMEEKNKIQEDLE 900

Query 900 KERFALQDLEGERENLIKRLNDIGLEAQECELREIETRDKCSKLEIEKKKLNEEIGNLSQ 959

KERFALQD+EGERENLIKRL DI +AQECE RE+ET+DKC+KLEIEK KL EI LSQ

Sbjct 901 KERFALQDMEGERENLIKRLKDIESDAQECESREMETKDKCNKLEIEKNKLQNEISQLSQ 960

Query 960 NLELEEQHLQKIQTEKLTADKRIKELEEKVAELEDKLNKLIREKKSLEEKLADIMSTLTE 1019

NLE EEQ+LQK Q +KL+ADKRIKELE+KVAELEDKLNKL +EKKSLEE+LAD+MSTL E

Sbjct 961 NLESEEQNLQKAQADKLSADKRIKELEQKVAELEDKLNKLTKEKKSLEERLADVMSTLAE 1020

Query 1020 EEKKSKQLMNLKSRHEASISELEERLSREQAARQDLEKTKRRLETELAEKSDNLSTQSHT 1079

EE+KSKQL LKSRHE SI+ELEERLSREQAARQDLEKTKRRLETEL+EKSDNLS+QSHT

Sbjct 1021 EEEKSKQLTKLKSRHETSITELEERLSREQAARQDLEKTKRRLETELSEKSDNLSSQSHT 1080

Query 1080 YEEIRITLEKAEIQITEMQTKLEEETMAKSAAQRQIRDVENAISEIKEDLEAEKRSKERA 1139

YEEIRITLE+AE QI +MQ KL+EET AKS AQRQIRD EN I+EIKEDLEAEKRSKERA

Sbjct 1081 YEEIRITLERAETQIIQMQAKLDEETTAKSLAQRQIRDAENVITEIKEDLEAEKRSKERA 1140

Query 1140 EKAKRDLAEEIESLKMELLDTGNTSEEQQTILRKKEVELQNIKKSLDDETKRAEAEIQEI 1199

EKAKRDLAEEIESLKMELLDTGNTSEEQQ++LRKKEVELQN KKS +DETKR E E+QEI

Sbjct 1141 EKAKRDLAEEIESLKMELLDTGNTSEEQQSVLRKKEVELQNFKKSFEDETKRTELEMQEI 1200

Query 1200 RKKAAVSIENLNNQLETSNKSKVAIEKAKLQLQEELNDANNELSILRSSKAESEKLKKNT 1259

RKKAAV+IENLNNQ+E +NKSK+ +EKAKLQLQEELNDANNEL+ LRSSKA+SEKLKKNT

Sbjct 1201 RKKAAVTIENLNNQIEAANKSKLVVEKAKLQLQEELNDANNELANLRSSKADSEKLKKNT 1260

Query 1260 EQQLSESLNRLAEVEEYKLDIETKLKKQTEVCDKLALSLESSESKLSQITKSEISLKSQL 1319

E QLSE +RL+E EE+KLD E+KLKKQTE+ DKLA++LE ESKLSQ+TKSE+S++SQL

Sbjct 1261 ELQLSEISHRLSESEEHKLDAESKLKKQTELADKLAMNLEEIESKLSQLTKSELSVRSQL 1320

Query 1320 EDLQKNFEEETRLKLAAQTSLRQVIGEISQIKDQLEEEQQMKENLEKHVQILQTQMQEVK 1379

DLQK+FEEETRLKL AQT+LRQ+ + + +KDQLEEEQQ KENLE HVQ LQ+QMQ+VK

Sbjct 1321 ADLQKDFEEETRLKLNAQTTLRQLESQFALLKDQLEEEQQSKENLESHVQALQSQMQDVK 1380

Query 1380 KKVEDDANQLLELDDSRKKIMREREELQIRLEEALNQVDRSEKAKRIIQAELQDAYHALE 1439

KKVE+DA QLLE D+SRKK+MREREELQIRLEEA+NQ +RSEKAKR IQAELQDAYHALE

Sbjct 1381 KKVEEDAQQLLEFDESRKKVMREREELQIRLEEAINQTERSEKAKRTIQAELQDAYHALE 1440

Query 1440 GQKNDQTQSDRKLKKMESQVNEIMNLNKKYLSEKEILEKENREKETRIMQLQREITANED 1499

QKNDQTQSDRKLKKMESQVNE +NLNKKYL+EK+ LEKENREKET+IMQLQRE+ AN+D

Sbjct 1441 SQKNDQTQSDRKLKKMESQVNETLNLNKKYLAEKDTLEKENREKETKIMQLQREMNANDD 1500

Query 1500 KLIELENSKSLLTRQLNELVSNKDDVGKNIGSLEKVKIQLETDLDNCKNTIEELEEENAT 1559

KL ELEN+KSLLTRQLNELVS+KDDVGKN+GSLEK KIQLETDL+NCK TIEELEEENAT

Sbjct 1501 KLSELENAKSLLTRQLNELVSSKDDVGKNVGSLEKAKIQLETDLENCKVTIEELEEENAT 1560

Query 1560 LQMNKERIEMQLTATKAQLDRELSSKDEMHDEQFKILNKKLRDTEATLEEEQKQRTSLAS 1619

LQMNKER+EMQLTATKAQLDREL+SKDEMHDEQF+I+NKKLRD EA LEEEQK +T++ S

Sbjct 1561 LQMNKERVEMQLTATKAQLDRELASKDEMHDEQFRIINKKLRDAEAALEEEQKSKTAMVS 1620

Query 1620 LKKKLEIENVDLMNQLSESEKIKEDLSRQIKKHQGTSINSQREMQEALNAKNEALKQIKE 1679

LKKK+E EN+DL+NQL+ESEK+KE+L+RQIKK+QG S N QREMQE + AKN+ LKQIKE

Sbjct 1621 LKKKIENENMDLLNQLTESEKVKEELARQIKKYQGNSSNLQREMQELVAAKNDYLKQIKE 1680

Query 1680 LEKRLRTADNSINQLTEDLSQLERVKRTIITERDDALEEIATLTVNKDTAIADKKRIESN 1739

LEKRLRT+++++NQL+EDLSQ ER KRTI +ERD+ALEEIAT+T+ +DTAIADKKRIES+

Sbjct 1681 LEKRLRTSESNVNQLSEDLSQSERAKRTIASERDEALEEIATITMARDTAIADKKRIESS 1740

Query 1740 LSALNMDYEELQLLLAELEDKNKKITNQLEQVQNELNVERQNVTRLENQKSTAERLNKEL 1799

+SAL+MD EELQLLL+ELEDKNKKI +QLEQVQNELNVERQN++RLENQKSTAERLNKEL

Sbjct 1741 ISALSMDNEELQLLLSELEDKNKKIISQLEQVQNELNVERQNISRLENQKSTAERLNKEL 1800

Query 1800 HEKIEELEVEAGKKYKTTVAAQQNKIQSLDAQLDAKSEEINQANRNNKKLERKLKEIMAL 1859

H+KIEELE+EAGKKYK TVAAQQ+KIQSLD+QLDAK EEINQANRNNKKLERKLKEIMAL

Sbjct 1801 HDKIEELEMEAGKKYKATVAAQQSKIQSLDSQLDAKIEEINQANRNNKKLERKLKEIMAL 1860

Query 1860 MVDEQKRADNAIEQIEKATGRYKKAERSLQMEQENNSALTTQNRRLQRDLEEVNETKEAR 1919

M DE KRADNAIEQIEKATGR+KKAERSLQMEQENNSAL+TQNRRLQRDLEEVNETKEAR

Sbjct 1861 MADETKRADNAIEQIEKATGRFKKAERSLQMEQENNSALSTQNRRLQRDLEEVNETKEAR 1920

Query 1920 DQEIKVLKTKIERLERRVRTGGRPNRGDAVSDDGLSGDGESLTDSGLGAVQQD 1972

DQEIK+LKTKI+RLE+R R GGRP RGD SDDGLSGDGES+TDS L +VQ+D

Sbjct 1921 DQEIKILKTKIDRLEKRTRAGGRPLRGDGGSDDGLSGDGESVTDSALKSVQED 1973

**Pairwise blastp: comp131819_c0_seq1 vs. SMU15000136**

Score Expect Method Identities Positives Gaps

786 bits (2031) 0.0 Compositional matrix adjust. 371/470(79%) 418/470(88%) 4/470(0%)

Query 25 LLFQLIPEKWLVDRNQVLLISMDGFRHDYIEKVKANGGKTPNFDYLISQGTRIMRSQNAF 84

+LFQ +P +W +DRNQVLLISMDGFRHDYIEKVKA GGKTPNFDYLI QG+RIMR++NAF

Sbjct 22 VLFQFLPSQWFLDRNQVLLISMDGFRHDYIEKVKAAGGKTPNFDYLIEQGSRIMRTKNAF 81

Query 85 PTITLPNHQTIVTGLYPENHGIVLNTFYDK---NETFDMNNQNNLNDKKWFEKWPEPIWV 141

PTITLPNHQT+VTGL+PE+HGIVLNTFYDK NETFDMNNQNNLND KWF+KWPEPIWV

Sbjct 82 PTITLPNHQTLVTGLFPESHGIVLNTFYDKTFPNETFDMNNQNNLNDPKWFDKWPEPIWV 141

Query 142 TLEKLGRLTASHLWPLTDAPIRGKLPFFQESQYTLLDNPPNYFPFMKRVEDVVNWLSNTR 201

TLEKLGRLTASHLWPLTD+P+ GK+PFFQESQ+TLLDNPPNYFPFMKRVEDVV WLSNTR

Sbjct 142 TLEKLGRLTASHLWPLTDSPVHGKIPFFQESQFTLLDNPPNYFPFMKRVEDVVYWLSNTR 201

Query 202 VHIDFSMLYFPEPDETGHGYGPNDQHVFDVVMKLDAVMGHLIQLLKSKGLWDRVNIILTA 261

VHIDFSMLYFPEPDE+GH YGPN Q F+ V+ LD+V+GHLIQLLK+KGLWD+VNIILTA

Sbjct 202 VHIDFSMLYFPEPDESGHSYGPNGQGTFNAVIMLDSVLGHLIQLLKAKGLWDKVNIILTA 261

Query 262 DHGMSENSFSRQIPLDTYVNPSWYSYTLLTPVGALYPVKGKEQLVYNALKNKNPHLQVFW 321

DHGMSENS SRQIPLD YVNPSWY+YTLLTPVGALYPVKGKEQ VYNALKN+NPHLQVFW

Sbjct 262 DHGMSENSDSRQIPLDEYVNPSWYTYTLLTPVGALYPVKGKEQAVYNALKNRNPHLQVFW 321

Query 322 REEVPRSLNYNIDNGRIAPLILIAENKWAISHKASNSTTIYGNHGYNNSEPDVQPFFIAQ 381

R+EVP SLNYNIDNGRIAPLILIAENKWAI+HKA+N T +YGNHGYNNSEPDVQPFFIAQ

Sbjct 322 RQEVPASLNYNIDNGRIAPLILIAENKWAIAHKATNGTNVYGNHGYNNSEPDVQPFFIAQ 381

Query 382 GPNIRSNYTIPFGYSVDIYPLMCYLLGIKPNPNNGSFERIKSLIEPKSIRNYDNTILYMK 441

GPNIR N+TIPFG+SVD+YPLMCYLLGI PNPNNGS ERIK +IEPKSI+ + ++YMK

Sbjct 382 GPNIRRNFTIPFGHSVDVYPLMCYLLGINPNPNNGSLERIKEIIEPKSIKKLCSVLIYMK 441

Query 442 YDIWITWTCIAIFGISFIIIIVVHIQSVRKKR-LSTKLKSAFAYSKIDSF 490

W+ T IA+F + II+ ++I KK+ + +K +AYSKI+SF

Sbjct 442 NSFWLLCTSIAVFWLCVIILFSLNICKCNKKKDVEENMKPTYAYSKINSF 491

**Pairwise blastp: comp141188_c2_seq3 vs. SMU15033813**

Score Expect Method Identities Positives Gaps

941 bits (2432) 0.0 Compositional matrix adjust. 465/683(68%) 566/683(82%) 12/683(1%)

Query 1 MALDIDLDIFRSKLADCKDRSQNSHFGVTSRALVAEEHRQKLILQELRELESKFKFRDDR 60

MALDIDLD+FR++LADCKDRSQN HFGV+S++L EEHRQ+LILQELRELE+ K++DDR

Sbjct 1 MALDIDLDVFRNRLADCKDRSQNLHFGVSSKSLKVEEHRQQLILQELRELENNLKYKDDR 60

Query 61 TSKTAAQISEIKDEIDSNISAMKYLGGISNMENLYSELTGNIGACVVHHSVNEYIPVNTL 120

T++TAAQI +IKDE++S IS++KYLGGISN+ENLYSELTGNIGAC +H SVN+ N L

Sbjct 61 TNRTAAQILDIKDEVESGISSLKYLGGISNLENLYSELTGNIGACALHQSVNQNFSENML 120

Query 121 ASKPLSSNINECWNYITKLSQLSQVHLRNASHYHQFYHDLNEISALLEKRWKIIANQISN 180

+K LS+N+N+CWNYITKL+ LSQVHLRNAS YHQF+HD+NEISALL+KRWK+I+NQI+N

Sbjct 121 VNKSLSNNVNDCWNYITKLAYLSQVHLRNASQYHQFHHDINEISALLKKRWKVISNQIAN 180

Query 181 FEPKGTIKESEQLANYLKDHLSYFMHLRNRIDNLQSESKRIVPIHLRQDPVSQNKLVGHS 240

FEPKGTIKES+QLAN LKDH SYF+HL RIDNLQ ESK+IVPIHLRQDPVSQNK++G

Sbjct 181 FEPKGTIKESDQLANNLKDHQSYFLHLWTRIDNLQIESKKIVPIHLRQDPVSQNKVLGQC 240

Query 241 LCSYSSPEISVKDHEEVFILNNSHPTTWKIQNSRGEIGEIPSVCIWIPGPDKLSSDKAIS 300

L SYSSPEISV D EEV ILNNS+P TWKIQN++GE+GEIPSVCIWIPGPD +SS+K IS

Sbjct 241 LYSYSSPEISVHDREEVVILNNSNPLTWKIQNNKGEVGEIPSVCIWIPGPDVISSEKPIS 300

Query 301 LQTQLIKNWEQCLNRFSIVLIDHYISLFERLLTDPVVCVNRSDPFNKLISDVQEILLSNP 360

L+TQL KNWEQCLNRFSIVLIDHYI+LFERLL +P + V++ D FN L+ DVQE+LL N

Sbjct 301 LRTQLTKNWEQCLNRFSIVLIDHYINLFERLLNEPNISVSKIDYFNNLLRDVQEVLLMNL 360

Query 361 TDMKSSELSGLLNELRKKLMLKHSEDSIATHLYTESETIKMHTPLIKYIDHLKNLRSLRD 420

TD+KS EL LL EL+K++ ++S DS +H+Y ESE IKMHTP+++YIDHLKNLRSLRD

Sbjct 361 TDVKSKELGALLVELQKRVNSRNSNDSRNSHVYKESEIIKMHTPMLRYIDHLKNLRSLRD 420

Query 421 SIQFNDYHQHN--RETKEKLYNSNLESLNKLYSENKHDLNKLLQSVDSIPNETYRHRHSQ 478

S+QFN N RET +K Y+SNL+ LNKL+S+NK++LN+LLQS++S+PNETY+HRHSQ

Sbjct 421 SVQFNQMDSQNISRETGDKRYSSNLDMLNKLHSDNKNELNRLLQSIESMPNETYKHRHSQ 480

Query 479 EIVHNSRSRDHINRDHRQFLSSSDLNHSHNYHLKSNHPEHHFAYKHD--HLTPDTYSPSV 536

E +H+S+ DH NRDHRQ+LS+SDL+HSH+YHL S+H E H K D ++ + +SPS

Sbjct 481 EFIHHSKFHDHTNRDHRQYLSASDLSHSHHYHLNSSHLESHTQLKPDRRNVHSEVFSPST 540

Query 537 SLTTSDIQLNRMNSPDYREFSNVRSIDNLTRNDNEEFHEYDDKLK-SRTLPLVLNS---E 592

SL+ SD QLNR D R+ N S DN N+ E EY + +K S +LP+ LN+ +

Sbjct 541 SLSVSDAQLNRH---DARDAHNFYSSDNFIDNEAELNQEYSEHVKASYSLPMALNNTDIQ 597

Query 593 EIKPKSHH-KKKKTWKVDSQTQLGVTVRDSKIQCNPTMYDESTFIEDEIFSTTDSSVTSS 651

IK KSHH KKKTWKVDSQTQLGVTVRDSKIQCNPTMYD STFI+DEIFSTT SS+ SS

Sbjct 598 SIKSKSHHPPKKKTWKVDSQTQLGVTVRDSKIQCNPTMYDVSTFIQDEIFSTTGSSIASS 657

Query 652 KQWSKSKAQKRYHLDAQTQIGVL 674

KQ+SKS + ++Y LDAQTQIGVL

Sbjct 658 KQFSKSMSPQKYQLDAQTQIGVL 680

**Pairwise blastp: comp145670_c2_seq3 vs. SMU15005691**

Score Expect Method Identities Positives Gaps

266 bits (679) 1e-85 Compositional matrix adjust. 140/321(44%) 198/321(61%) 8/321(2%)

Query 1 MSPNYMPNNLALTDYMRYWNTTDNLRCVYMISSRKQNSFTIKITAL-TYFWYNIWINNQN 59

MSPN++P + NL C Y +SSR SF++ AL + Y++ +NN

Sbjct 149 MSPNFLPKK-PALAALLNNANQSNLNCTYRVSSRTNKSFSVNFDALYGRYKYDLIVNNTY 207

Query 60 VNGLTKITFSGDQRVSVVFEAYYYWYYSWYYYNSLSGFTGAVDLAYDVLNLLPVDNVPFR 119

+ K+ GDQ++ + YY + S +L GFTG VD++Y + LLP+D P R

Sbjct 208 IYSPVKVAVPGDQKLEFILRTYYSYSDS-----NLFGFTGTVDMSYQSITLLPIDGNPLR 262

Query 120 GVPQISYQGVNYSVQYESLSPLDAQVMCLTLGAVGYKSQKPSLNFTDRKLVSPSYQCLGP 179

G+P +SY G NY++Q+ SLS D V+C TLG KS +P N T KL+ P++ C G

Sbjct 263 GIPLLSYLGENYTIQFGSLSTYDGHVLCQTLGYGSVKSMRPYKNETGTKLIYPTFNCRGS 322

Query 180 EKLLFHCLDLDVQKLFYKEFYNAPKYTTVLTCSEKFKLRLIDGDSSSSGLVQIKYGGIWS 239

E L C +LD +KL Y+++Y T +TCSE F ++L+ GD+ S G+VQ+ G +S

Sbjct 323 ESRLTQCGELDGKKLHYEKYYYTSPNVTNVTCSENFNMQLVGGDNRS-GVVQLNLGEGFS 381

Query 240 YMCTDNMDSSAADVICGELGYKSALYYESNSGNRNSSNYYNFPDGEYYKYRYGTVMNNIR 299

Y+C DNMD SAAD IC E+G++ +LYYE GN +SNYY++ G+YY+YRY +M NIR

Sbjct 382 YICADNMDPSAADAICMEMGFQGSLYYEGYMGNTGASNYYDYFSGDYYRYRYDFIMTNIR 441

Query 300 CNSETKLKNCYYQTSNNYYNY 320

CNS TKL+NC YQ S++YYNY

Sbjct 442 CNSNTKLRNCKYQISHSYYNY 462

**Pairwise blastp: comp146596_c0_seq1 vs. SMU15025275**

Score Expect Method Identities Positives Gaps

452 bits (1163) 6e-168 Compositional matrix adjust. 225/241(93%) 233/241(96%) 0/241(0%)

Query 1 MTERDENVYKAKLSEQAERYDEMVKSIKLVAESGVELTVEERNLLSVAYKNVIGSRRSSW 60

MT+RD+NVYKAKLSEQAERYDEMVKSIKLVAESGVELTVEERNLLSVAYKNVIGSRRSSW

Sbjct 14 MTDRDDNVYKAKLSEQAERYDEMVKSIKLVAESGVELTVEERNLLSVAYKNVIGSRRSSW 73

Query 61 RILSSIEQKEESKATNTNRIEIARNYRKQVEKELSGICGEVIKILDDHLIKTATTTDSKV 120

RILSSIEQKEESKA +T+RIEIARNYRKQVEKELSGIC EVIKILDDHLIK A+TT+SKV

Sbjct 74 RILSSIEQKEESKAISTSRIEIARNYRKQVEKELSGICIEVIKILDDHLIKVASTTESKV 133

Query 121 FYLKMKGDYYRYNAEFSIEKDRQEIAEKSNQAYTEALDLSDELGATHPIRLGLALNYSVF 180

FYLKMKGDYYRYNAEFSIEKDRQEIAEKSNQAY+EAL LS+ELGATHPIRLGLALNYSVF

Sbjct 134 FYLKMKGDYYRYNAEFSIEKDRQEIAEKSNQAYSEALQLSEELGATHPIRLGLALNYSVF 193

Query 181 FYEIMGSSEKACELAKKAFDKAISELDSLTEEDSYKDSTLIMQLLRDNLTLWTADDDDQK 240

FYEIMGSSEKACELAKKAFDKAISELDSLTEEDSYKDSTLIMQLLRDNLTLWTADD

Sbjct 194 FYEIMGSSEKACELAKKAFDKAISELDSLTEEDSYKDSTLIMQLLRDNLTLWTADDGKNL 253

Query 241 N 241

N

Sbjct 254 N 254

**Pairwise blastp: comp141858_c0_seq6 vs. SMU15002375**

Score Expect Method Identities Positives Gaps

911 bits (2354) 0.0 Compositional matrix adjust. 432/443(98%) 440/443(99%) 1/443(0%)

Query 1 MREIVHLQAGQCGNQIGSKFWEVISDEHGIDPTGTYHGDSDLQLERINVYYNEATGGKYV 60

MREIVHLQAGQCGNQIGSKFWEVISDEHGIDPTGTYHGDSDLQLERINVYYNEATGGKYV

Sbjct 1 MREIVHLQAGQCGNQIGSKFWEVISDEHGIDPTGTYHGDSDLQLERINVYYNEATGGKYV 60

Query 61 PRAVLVDLEPGTMDSVRAGPFGQIFRPDNFIFGQSGAGNNWAKGHYTEGAELVDSVLDIV 120

PRAVLVDLEPGTMDSVRAGPFGQ+FRPDNFIFGQSGAGNNWAKGHYTEGAELVDSVLD+V

Sbjct 61 PRAVLVDLEPGTMDSVRAGPFGQLFRPDNFIFGQSGAGNNWAKGHYTEGAELVDSVLDVV 120

Query 121 RKEAESCDCLQGFQLTHSLGGGTGSGMGTLLISKIREEYPDRVMLTFSVVPSPKVSDTVV 180

RKEAESCDCLQGFQLTHSLGGGTGSGMGTLLISKIREEYPDRVMLTFSVVPSPKVSDTVV

Sbjct 121 RKEAESCDCLQGFQLTHSLGGGTGSGMGTLLISKIREEYPDRVMLTFSVVPSPKVSDTVV 180

Query 181 EPYNATLSVHQLVENTDETFCIDNEALYDICFRTLKLTTPTYGDLNHLVSATMSGVTTCL 240

EPYNATLSVHQLVENTDETFCIDNEALYDICFRTLKLTTPTYGDLNHLVSATMSGVTTCL

Sbjct 181 EPYNATLSVHQLVENTDETFCIDNEALYDICFRTLKLTTPTYGDLNHLVSATMSGVTTCL 240

Query 241 RFPGQLNADLRKLAVNMVPFPRLHFFMPGFAPLTSRGSQQYRALTVPELTQQIFDAKNMM 300

RFPGQLNADLRKLAVNMVPFPRLHFFMPGFAPLTSRGSQQYRALTVPELTQQ+FDAKNMM

Sbjct 241 RFPGQLNADLRKLAVNMVPFPRLHFFMPGFAPLTSRGSQQYRALTVPELTQQMFDAKNMM 300

Query 301 AACDPRHGRYLTVAAIFRGRMSMKEVDEQMLNVQNKNSSYFVEWIPNNVKTAVCDIPPRG 360

AACDPRHGRYLTVAAIFRGRMSMKEVDEQMLNVQNKNSSYFVEWIPNN+KTAVCDIPPRG

Sbjct 301 AACDPRHGRYLTVAAIFRGRMSMKEVDEQMLNVQNKNSSYFVEWIPNNIKTAVCDIPPRG 360

Query 361 LKMSSTFIGNSTAIQELFKRVSEQFTAMFRRKAFLHWYTGEGMDEMEFTE 410

LKMS+TFIGNSTAIQELFKRVSEQFTAMFRRKAFLHWYTGEGMDEMEFTE

Sbjct 361 LKMSATFIGNSTAIQELFKRVSEQFTAMFRRKAFLHWYTGEGMDEMEFTE 410

**Pairwise blastp: comp135193_c0_seq1 vs. SMU15002770**

Score Expect Method Identities Positives Gaps

1236 bits (3197) 0.0 Compositional matrix adjust. 583/634(92%) 612/634(96%) 0/634(0%)

Query 4 LIWEGDNEIPQEVHEVVIPHLGHMPICKGDYHYLPKAVQGFIAKWALICKPRALYICDGG 63

+IWE D EIPQEVHEVVIPHLGHMPICKGDYHYLPK VQGFIAKWAL+CKPRALYICDGG

Sbjct 1 MIWENDTEIPQEVHEVVIPHLGHMPICKGDYHYLPKKVQGFIAKWALVCKPRALYICDGG 60

Query 64 EDEASEIITKLKERGTLHALTKLENCYICRTDPDDVARVESKTFISTKDKHMTVPHVKEG 123

EDEA+EIITKLKERGTLH+LTKLEN +ICRTDPDDVARVESKTFISTK+KHMTVPHVKEG

Sbjct 61 EDEATEIITKLKERGTLHSLTKLENSFICRTDPDDVARVESKTFISTKNKHMTVPHVKEG 120

Query 124 STGILGQWMAPESLSEEVAQRLRGCMAGRMMYIIPFSMGPIGSPLSKIGIQLTDSNYVVL 183

S GILGQW+APE L+EEVA+RLRGCM GRMMYIIPFSMGPIGSPLSKIGIQLTDSNYVVL

Sbjct 121 SKGILGQWIAPEDLNEEVAERLRGCMGGRMMYIIPFSMGPIGSPLSKIGIQLTDSNYVVL 180

Query 184 SMRVMTRVSPLVWHYLGDKAFVKCVHSVGCPRPHQRKVVNHWPCNPEKTLIAHLPDERLI 243

MR+MTRVSPLVWHYL D AFVKCVHSVGCPRPH RKV+NHWPCNPEKTLIAHLPDERLI

Sbjct 181 CMRIMTRVSPLVWHYLSDNAFVKCVHSVGCPRPHPRKVINHWPCNPEKTLIAHLPDERLI 240

Query 244 ISYGSGYGGNSLLGKKCFALRIAGRIAYDEGWMAEHMLIMSVTNPKGEEKFIAAAFPSAC 303

ISYGSGYGGNSLLGKKCFALRIAGRIAYDEGWMAEHMLIMSVTNPKGEEKFIAAAFPSAC

Sbjct 241 ISYGSGYGGNSLLGKKCFALRIAGRIAYDEGWMAEHMLIMSVTNPKGEEKFIAAAFPSAC 300

Query 304 GKTNMAMLEPSLPGWKVQVVGDDIAWMRFDEHGELRAINPEAGFFGVAPGTNYKTNPNAM 363

GKTNMAMLEP LPGWKVQVVGDDIAWMRFDEHG LRAINPEAGFFGVAPGTNYKTNPNAM

Sbjct 301 GKTNMAMLEPGLPGWKVQVVGDDIAWMRFDEHGVLRAINPEAGFFGVAPGTNYKTNPNAM 360

Query 364 KTFQKNSIFTNVAETSDGSYYWEGLEDEIDKNLEITSWLGVKMRVGDKSKGLAAHANSRF 423

KTFQKNSIFTNVAET+DGSYYWEG+E E+D NLE+TSWLGVKM+VGDKSKG AAH NSRF

Sbjct 361 KTFQKNSIFTNVAETNDGSYYWEGMEKEVDPNLEVTSWLGVKMKVGDKSKGPAAHPNSRF 420

Query 424 CCPASQCPIIHPKWEDPKGVPISAIVFGGRRPEGIPLVMQAFNWKHGVMLGASLKSEATA 483

CCPASQCPIIHPKWEDP+GVPISAIVFGGRRPEGIPLVMQAF+WKHGVMLGASLKSEATA

Sbjct 421 CCPASQCPIIHPKWEDPQGVPISAIVFGGRRPEGIPLVMQAFDWKHGVMLGASLKSEATA 480

Query 484 AAEFKGKQIMHDPMAMRPFMGYNFGKYLQHWLDLEKPGRKMPLIFHVNWFRLNDKGKFVW 543

AAEFKGKQIMHDPMAMRPFMGYNFG+YLQHWLDLEKPGRKMPLIFHVNWFRLN++GKFVW

Sbjct 481 AAEFKGKQIMHDPMAMRPFMGYNFGRYLQHWLDLEKPGRKMPLIFHVNWFRLNEQGKFVW 540

Query 544 PGFGDNIRVIDWMCRRVNGEDIAEPSPIGLIPKHGTINVTGLDAKWEEMFALPKTYLAED 603

PGFGDNIRVIDWMCRRVNGEDIAEPSPIGL+PK G+++++GLD KWEEMF LPK YLAED

Sbjct 541 PGFGDNIRVIDWMCRRVNGEDIAEPSPIGLLPKKGSLDISGLDVKWEEMFNLPKNYLAED 600

Query 604 IQETVKFLREQTGEDLPAGVIQELNEQEKRIHNM 637

IQET KFLREQTGEDLPAGVIQ+L+EQEKRIHNM

Sbjct 601 IQETKKFLREQTGEDLPAGVIQQLDEQEKRIHNM 634

**Pairwise blastp: comp86205_c0_seq1 vs. SMU15039048**

Score Expect Method Identities Positives Gaps

297 bits (760) 2e-110 Compositional matrix adjust. 149/149(100%) 149/149(100%) 0/149(0%)

Query 1 MADQLTEEQIAEFKEAFSLFDKDGDGTITTKELGTVMRSLGQNPTEAELQDMINEVDADG 60

MADQLTEEQIAEFKEAFSLFDKDGDGTITTKELGTVMRSLGQNPTEAELQDMINEVDADG

Sbjct 1 MADQLTEEQIAEFKEAFSLFDKDGDGTITTKELGTVMRSLGQNPTEAELQDMINEVDADG 60

Query 61 NGTIDFPEFLTMMARKMKDTDSEEEIREAFRVFDKDGNGFISAAELRHVMTNLGEKLTDE 120

NGTIDFPEFLTMMARKMKDTDSEEEIREAFRVFDKDGNGFISAAELRHVMTNLGEKLTDE

Sbjct 61 NGTIDFPEFLTMMARKMKDTDSEEEIREAFRVFDKDGNGFISAAELRHVMTNLGEKLTDE 120

Query 121 EVDEMIREADIDGDGQVNYEEFVTMMTTK 149

EVDEMIREADIDGDGQVNYEEFVTMMTTK

Sbjct 121 EVDEMIREADIDGDGQVNYEEFVTMMTTK 149

**Pairwise blastp: comp142073_c0_seq1 vs. SMU15038409**

Score Expect Method Identities Positives Gaps

1004 bits (2595) 0.0 Compositional matrix adjust. 501/934(54%) 683/934(73%) 12/934(1%)

Query 1 MMKLNFFLILLLSIVRNYCLCNENEQWTSENEYSNYQVETLQKITDWDCWYKDYYLKVSH 60

M F I++ ++ N C +E QW ++ S+Y +E +ITDWDCWYKDYY KV

Sbjct 4 MTVTKFISIIIFLVISNSC-TSEEVQWNEKDNNSDYNIENSNRITDWDCWYKDYYQKVDR 62

Query 61 CPSKTLKSKEKIKCKQYKDCKRYCSSVYGIEQCRVRCQSKKTDLSKTIKVIYRVRKDGEC 120

C SKT+ S E IKCKQY+ CKRYC+S+YG+EQCRV C K +L+K IKV+YR+R++ C

Sbjct 63 CHSKTISSTEHIKCKQYRGCKRYCTSIYGLEQCRVHCTLKTENLTKYIKVVYRIRQNENC 122

Query 121 KDSVICVYRTKCQGLWPKYIHQGCYYTGEFKSWWLKHKKEYAKPRIY--GQIQPSGKIYN 178

K+SVICVY+++C GLWPKYI+QGCYYT F WWL+H+KEYA+PRI I P+ IY+

Sbjct 123 KNSVICVYKSECNGLWPKYINQGCYYTQAFSQWWLEHRKEYARPRISKRSTIPPTKNIYS 182

Query 179 CIINNARFPLINPSLETTVQFRMSDNKKLFYKIGQKWFKAKFVVGNLFFWIGKLWVPVRF 238

C+ N R+P I+PS E +QFRMS K ++YKIGQ WF+AK V NLFF IG+ VPV+F

Sbjct 183 CLNNMGRYPKIDPSSEIVIQFRMSRGK-MYYKIGQVWFEAKLVDRNLFFLIGEKQVPVKF 241

Query 239 SRKHQYWKIGDKWIQVIVLRNTVYSNINSSWIPLKVMFGKKYFKIGKRWLNLKITNNKIH 298

+RKH YWKIG+KW+++ LR +Y +I ++WI LK+ GK+YF++G++W+ L+ +N K+

Sbjct 242 TRKHHYWKIGEKWLKIAALRQKIYGHIQNAWIELKISSGKEYFRLGRKWVRLQFSNRKVE 301

Query 299 YKFGGKWIEIKILQNNLYNKINGVWYKVKFHKNFNLIKIGQSYRKIKSIHEIIYMKLGKR 358

YK G KWIEIKILQN Y KI+G WY+VKF +FNLIKI +YRKIK I + Y+KL KR

Sbjct 302 YKCGKKWIEIKILQNYFYGKISGKWYRVKFGNHFNLIKIKGNYRKIKFIEGLNYIKLAKR 361

Query 359 WFEFTVENKRYQIRFDKKWVEIIFDKGLVFFLIDNNKYQIRFKFYSMFIELNKKWFQIEF 418

WFEF ++ +Y I+FD+KW+EI FDK F LI++ KY + FK S+++++N +W Q+E+

Sbjct 362 WFEFEIKKGKYFIKFDQKWIEIEFDKLQNFVLINDIKYPVLFKLMSVYVQINNQWIQVEY 421

Query 419 QKEKLFLKTNKIWLHILLKENHFYIKYNGKERKLIVDENRNIFYKSSLKDKLLSLDEKEI 478

+ ++F K NK W+H L EN + + N K K+ ++ RNI Y+SS K+ ++

Sbjct 422 KNNEIFFKVNKNWMHFSLIENRLFFRKNKKLVKISLNSKRNILYRSSSSGKMKPFKNSDL 481

Query 479 KSGNSFPIDQFIKKENSFSLKYFSGYWLPFKIEKGESLFFISGKWLNLKNENHKLFYKYQ 538

K G F +QF K ENS +K F+G+WLPFK E G LF +SG+WL LK E++KLFY+ +

Sbjct 482 KLGKYFSNNQFSKIENSLKMKLFNGHWLPFKKENGSFLFQLSGQWLKLKEEDNKLFYQTK 541

Query 539 EQWIEVCFIGKNEFNKIQGHWIQVKRDENRLFYFDSKFWHQIQLTGKYVAIKINDSWKSF 598

E+WIE IGK EFN++QGHW++VKR+ + ++FDS WHQI+L G++ A+K N SWKS

Sbjct 542 EKWIEFILIGKTEFNRLQGHWVKVKRENEKFYFFDSDSWHQIKLVGQHYALKFNGSWKSL 601

Query 599 AFVNGKKFIRLGNKFSEIKFNSENQNEFHLKIGGKWHNLKMENRNFFIEFNEKWTRVEFY 658

FVNGKKFIRLGN++S++KF+ + QN+F LK+GGK++ L + F+I+ N W +VEFY

Sbjct 602 GFVNGKKFIRLGNRYSQLKFSKKGQNKFSLKMGGKFYKLMSKENLFYIKLNNDWKQVEFY 661

Query 659 SHRSMNILFDNNWLSVTFKNKKLYVKNENCWLETIFKTNKIFMKIGDTLTEMKISENKDF 718

HRS+N+L +N WL + FK++K Y+KN NCWLETIFK NKIF++IGD E+KIS +KD

Sbjct 662 PHRSINMLINNAWLPMAFKDRKFYIKNGNCWLETIFKGNKIFIRIGDAFLEIKISSDKDI 721

Query 719 KVKINGHFYKLIENNQIQYNKVILKISFINEKLHAFINGNWLRLRIDKENLMVKVIDSWI 778

VKING +KLIE N+I+ NK+ILK++FINEKLH F NGNW+ R ++E +KVIDSWI

Sbjct 722 LVKINGKSFKLIERNEIELNKLILKVNFINEKLHIFFNGNWVPFRFEEEKTFIKVIDSWI 781

Query 779 EAEIDENKQISVKVNNEKVILSFDNGIICETYENVKIPISIRFKQLYYNLDGKWIKIYLK 838

EA+IDE K I+VK+N+EK+ L+FDNGI+CE Y+++K+PI+IRFKQLYYNL+ KWIK+Y +

Sbjct 782 EAKIDEKKHITVKLNDEKIQLTFDNGILCENYDDLKVPINIRFKQLYYNLNEKWIKVYPR 841

Query 839 DNLRYFKENGNLVILDRDFLKNKTRKYKLKLMLKCTLLSDN------IKELTNFG--RSE 890

DNLR+ K N NLV LD+++L++K RKYKLKL LKC +LS N +KE N RS

Sbjct 842 DNLRFIKLNKNLVKLDKEYLRDKIRKYKLKLKLKCAILSRNQRHWTKLKERLNIESRRSV 901

Query 891 FKRNIQRNKHENMRYYQTIDMLKKPRQKSKQEII 924

F+R I RNK +N + Y+TIDM KP+ K E+I

Sbjct 902 FQRLIGRNKLKNTKIYETIDMRHKPKPADKNEVI 935
