# Supplementary material for: Planarian regeneration in space: Persistent anatomical, behavioral, and bacteriological changes induced by space travel
Source: Regeneration (Oxf). 2017 Jun 13;4(2):85–102. doi: 10.1002/reg2.79 (PMC5469732; doi:10.1002/reg2.79)
Supplement: Supplementary file 5 — Table S1: Proteins identified using mass spectrometry that were present in the space‐exposed worm water sample but not in the Earth‐only worm water sample [file REG2-4-85-s005.docx]

**Supplemental Table 1: Proteins identified using mass spectrometry that were present in the space-exposed worm water sample, but not in the Earth-only worm water sample**

| **Accession number for DNA sequence** | **Number of unique peptides** | **Sequence of identified peptides** | **Putative sequence of *D. japonica* protein (MS-identified peptides are in bold)** | **SmedGD ID of *S. mediterranea* homolog** | **Percent identity** | **Alignment length** | **Mismatches** | **Function in *S. mediterranea*** |
| --- | --- | --- | --- | --- | --- | --- | --- | --- |
| comp145442_c0_seq1 | 10 | [K].IINGDQFECSCSCEGER.[C]  [K].ISDSCLPGTCPLNADCEER.[L]  [K].TGGGCINTPGGFECTCSAK.[Q]  [K].GKPICSCEECTAADR.[T]  [K].SGYSQNSQGDCVDIDECK.[I]  [K].IGESQSGTFR.[S]  [K].ALQCQIGEECK.[L]  [K].DGYQEDQIGVCR.[N]  [K].SYNPCQQINECVK.[W]  [R].SCIDDNECALLK.[C] | MITDLPVGITVINLKPNPSICTNTSMPTNEKSINLTYLAVNSDNLNNLTNVSSHCEQFVSYDCQNSPITDGIKYGAYDNSGKYIEYWIGPKDKKFCANETCNCAVIDDKMRSDSGKFTDKTILPLSSISLPKVTGKRKLCINELRCYNLPKTCDEYKQQRRLDINKGNRNNIWAIDPDQAGGETYFGVLCKMIDSVTVSETLQESNPLSINSNNNKATTVNIPFVNTSPLQIQNLVNQSNYCSQRVDYLCKNSGSLYNNSPKLFDFKNNPIITWAGADLYSAVGSCACDVLGSCPLNSKCRCDALSNSTQYEGGIFTDRTILPIQRIQYQKGQSIQSNLYPVDCGSQPFDLPKDCIDARTKGYTYDTEILIKPNGVSKPYLVQCLMNGGKNKDLQITVVPVKVASIQNNESIPFIYPSAPFNDIKQLVQNSLYCIQPMKANCKGILFSSVFTWTDGNNTTQTTFGTFNKNTFCPCGLNHVCAGIPGESKQSMFNRKCNCDTPNYNVSLSEYVLISTRNILPIKSLNFKLSPDSDPMNSVTVGSLMCANTRFDFDECATSFHDCDSHANCTNLDYNYRCDCNSGWQGLGGNEMYSNGR**SCIDDNECALLK**CPSVSDCLNTPGSFICTCHIGFQKTAPTVCIDIDECKRNSSICDQNARCVNTYGSYLCTCNAGFRGSGKPGDCEAVATCGCWGDPHCLSFDGNWFHYQGRCKYTLVRDECENGLPKDSSTPNFEVIMKNWDQNTGKNSMVSWAKEITVKIMNYTIMMKIGLFLIVNGQKTSVPFNPKLNDGTNVGFEVAFFGSTLRLTCITGLEIKWDGVSRVDVTIPGRYMKNVCGQCGNYNLNPLDDWIVGPKCKPSGKTTDLLNLFGDSWRNDDPADTDPFCSSDTCNETPNEKPCDSALMTKSKLECEKLREKFLACEIVMRQMNHSLDEYIESCVYDQCYSTGDLTQMMCKTAESLAQKCLEEYKVKVLYRSVNFCNMVCNKNMVYSSCASPCQPTCYNNTNSMLCNGPCVESCVCVDGYVMENGICINPKSCGCLMSDGTYYSNGEQRTNEICSMKCGCNGKTGQLECSNITCSNDAFCDFKDNDYGCHCKNGFMGDGIICKDIDECSLIPSVCDINADCKNSIGSYSCSCKEGFEGNGKTCNNINECYPLSPCDNSTEECFDQIPGYQCRCKNGFLKNMTTGKCEDKNECADTGNLCDRVSTNCNNTFGSYRCDCKYGFRPSPIDSFVCTDVNECNLVHECDKNYAKCTNTPGSYYCTCDSGFQGDGRNCTDIDECITKKVCQRPDATCVNLPGSYECRCLNGKPGCDGDNPCNTVRCNHLNEICYLGQCFCKRGFERNITTNQCEDINECETAANDCAERIAKCVNLDGSYECECLYGYRMSPVNNSCENINECAEDLHKCKENAKCVDTKGGYYCECQTGFTGHCDECRDIDECVLSLHKCDQDYGTCENSIGSYVCRCNEGYSGDGSICSDINECLIGVHNCSNLNQYCLNVDGGYECKCLSGYKMDIDGTCSDINECSFSDNGCDDVAHCENIQGSFRCNCPNGYTGNGKTCTPIDGHEKCGRLLCPLNAICENSTCQCKHGYKNSSNSCENINECKNQTSCDVNANCIDTHGSFICYCNEGYVGDGYNCYKKTTEIDICDTDSFCIDGDCYDGECHCPQGYVFKANKCIDERCKNVCPKTAICSISSGSPVCSCSVGTSLSSDNSECIDIDECRDNSHDCQMNSFCLNRFGSYDCKCPKNNVDVFNDGKICIAVQPAKCDHECPIGQYCNSGNCSCLPGLNAKLDNEHLICNK**ISDSCLPGTCPLNADCEER**LFGYLCKCKAGYEGVGVKSCRDIDECGSKTDNCTINENCNNIPGSFNCLCKKGYSRNSTTGFCETNNKCNCGPHGICGNDSKCLCKPGYQVNEKGVCEDVDECQTQSPCHMLAQCTNTLGSYKCSCPENFYGDPENKCIENKCKTK**ALQCQIGEECK**LSLYGSVCQKKICNSTEILVDNECLPVNTLCQAVSCGTNAVCKIENGRPDCFCDSGYSGDGEKCSDIDECSNGLVNCPNNSFCQNKDGSYHCACNIGFQRPGNSTASDPCTDIDECVIPQSCATNALCENSIGSFRCECKEGYIGDGKYSCQIDSKCKRHGGCHEKAACILIAEKSSYTCQCNSGYYGDGINNCIKNNLCFENGQSKCHENAICQQLNATYQCICPSGFLGDGYNFCNDINECSNKNSHNCSKLEKCVNLDGDYKCVCVDGADIVNNVCVDIDECSSNTTNKCSSDAVCKNKIGTYTCQCKDGFFGNGILCHDIDECKRGLANCSENALCINKPGSFACECQAGTIGNGTTCEDEDECKKPKGTSGAAECDENAVCKNKDPGYTCVCNSGYKGTGWFCVKETPCDKPNACSSNQTCTPSDDNSLAICECKPEFKLENNNCVSKQECDNDIDTCDRQTSNCIQLDRGFKCECKPGFKMVGNTCQDKNECDPTSTDFAAGECFK**TGGGCINTPGGFECTCSAK**QETQENNSCKPIDSCALKLDKCDKKVENCISNKDSSYTCKCIEGFEKVDNHCVDIDECANKIDSCKNNATCTNTIGSYTCNCPENLKLDTSKKSCTDRNECKENPMICGELSKCLNTDGSYKCVCVTGYKWNGTHCEDIDECANKTHPCHEFAQCSNVPGSCKCKCKQGYTGDGIYSCQEIDKCPSREDVKCPVGTYCNTIGNIVYCNCTSGYEDSQNMECIGDVCYKDCNDINECDTKKSACHELAKCTNNKGGYVCTCPSYLVGDGKISCKDQNECALEIHSCDVNTSYCENLDNSMDVLEPYQCHCFNGYVKIPGTQICVNKNECLNPAENDCVENSVCVDTVGSFECKCENGFKQLPTGKCVDIDECEEKLDNCSRNSVCVNRIGSYYCECNSGFHWSDNTYINCEDINECDNPTSCNLLSNSLCVNNPGSFFCLCQPGMSLQGGQCTDINECLKENSCGKNAKCTNTVSSYNCSCSDGLRKLPNGDCELINECLEGIDRCEELNGANSTCYDLPVGYGCVCPKGFVNDRKLAQICENQNECEINIHNCSVANSDCVDTYGSFKCECK**SGYSQNSQGDCVDIDECK**ISEKNQIDLCNTGKCVNTKGSYICECPSGFVNDNNNCTDVNECLLYNGTNESNNCDHENGYCINSEGSFACRCKEGYSMLENSTVCQDIDECNFTNSCLDGLCNNLPGSFECLCKKGYNLVNGKCEDVDECFNDSLKCGPEIAGVCLNKIGSYQCKCNVGYENSGGK**SYNPCQQINECVK**WNVTCPANAQCVDRSVGYECICK**DGYQEDQIGVCR**NINECKIDNTCHYRAKCVDTDGSYKCICKYGYKGDGKVSCKAICGPNSCPAGQLCK**IINGDQFECSCSCEGER**CRETGPVCNTRGMTYSSEKEMFESSCKLNISTEVEYYHACQQSCSKVVCPGNEKCTISK**GKPICSCEECTAADR**TPRVLCGSNGIEFKSVCQMKTWMCNSKTEISISYDGPCHKSIDCSVSEWTNWSSCSKTCGIGRSSRTRMVLKVALFNGECDEVLSETQQCYNGPCPGDECENISCPPSSFCEFGKCVCPDCSNQKTSDPVCAK**IGESQSGTFR**SFCTLLHNACMLNSTFTYLHKGICGDHVPSEPKICTMVTNFKIVQSNDNCTSTEPLRVNLCSGGCGKNPKYCCRPENNRILRARFICPDNSYVIRQVKSISSCECKLEENIL | SMU15038343 | 73.13 | 3718 | 996 | Putative *C. elegans* protein MUA-3 isoform 3 (fibrillin homolog) |
| comp128998_c0_seq1 | 3 | [R].GNDECGIESLGVASEPIL.[-]  [R].RGNDECGIESLGVASEPIL.[-]  [K].FAEDFGMAQENCNPYK.[G] | MILAILLSFCLVQLSVSDTPANCTYQDVIGKWTVYTGNFSVSCSTSQFVATKTLTLIYPNLAIDEFGNYGKWTLIYNQGFEVIINNKKYFGFFDYKKINSTYAISYCDRLQPNWFHDVLIRQWQCFKAQKVSSLKEKNNLSPNSNVFALTSLRFGSQKRIVDKINMGNNGWTAKDYPEFHEKTLYEITNMAGGSRSKLQRPKPAPITKSILESVKLIPKSFDWRNVNGVNYVSPVRNQGGCGSCYSFASAGMLEARYRIKSNNTILPILSPQDVVECSPYSQGCDGGFPYLIAGK**FAEDFGMAQENCNPYK**GVDGKCSTTKNCKRYFATNYKYIGGYYGATNEPLMRMELVRNGPIAVGFEVYNDFMGYRGGVYHHNFATRILTTSKFGFNPFELTNHAVLVVGYGETQSGEKFWTVKNSWGVGWGESGYFRIR**RGNDECGIESLGVASEPIL** | SMU15000643 | 82.12 | 453 | 81 | putative cathepsin C |
| comp127150_c0_seq1 | 3 | [K].ITNQLEQVQNELNVER.[Q]  [K].LQLQEELNDANNELSILR.[S]  [R].DVENAISEIKEDLEAEK.[R] | MVTIEAKNLQYLQVDKSLVSDSANLSDWANKKLVWIPDEIEGFISGSLIEEKGDEATVKLENGKSVKFPIENIQKMNPPKFLKSEDMADLTYLNEASVLFNLKDRYFSDLIYTYSGLFCVVVNPYKRLPIYTDNVIEWYKGRKRHERPPHIYAVTDVAYRNMLQDKENQSILCTGESGAGKTENTKKVIQYLASVATSLKNQKTTANNALSQFYAHDVNIGELETQLLQANPILEAFGNAKTIKNDNSSRFGKFIRINFDNSGFISSANIETYLLEKARVIRQAASERCFHIFYQLLIGADDNLKKELILENVSTYKLLSNGMITVPDYDERQMFKDTVESLDIIGISKDEQDSIFRVISAVLHMGNIEFKQERSSDQAALPDNTVAQKVAHLLGLPVTEMTKALLKPKLKVGREVVAKAQTKEQAEFSVEAISKSTYERMFRWLVMRINRSIDRNRQKTNFIGILDIAGFEIFEINSFEQLCINYTNEKLQQLFNHTMFILEQEEYRKENIKWEFIDFGLDLQPTIDLIEKPMGILSLLDEECFFPKATSKTFVEKIIKNQSSHSKFKATDFRAKADFGVIHYAGKVDYVADNWLVKNMDPLNENVVSLLQESNELFVQTIWKDTENIIGLSTTTAQESAFGGAKTRKGMFRTVGQLYKESLTKLMDVLNNTSPNFVRCIIPNHEKKAGKIDSRLVIDQLKCNGVLEGIRICRQGFPNRILFQEFKQRYGILTPNVIPKGFMDGRKATELMLGALDIDVSNYRIGQSKIFFKAGVLARLEEDRDTKLTEIIVKFQSFARGYLARKNLQNRSQHLNAVKIIQRNCTAYLKLRNWSWWKLFTRVKPLLSVTRQEEVVALKEEELKKTKESLEKMTNEFEVTKKNYESLTEEKSKLQEDLEKERFALQDLEGERENLIKRLNDIGLEAQECELREIETRDKCSKLEIEKKKLNEEIGNLSQNLELEEQHLQKIQTEKLTADKRIKELEEKVAELEDKLNKLIREKKSLEEKLADIMSTLTEEEKKSKQLMNLKSRHEASISELEERLSREQAARQDLEKTKRRLETELAEKSDNLSTQSHTYEEIRITLEKAEIQITEMQTKLEEETMAKSAAQRQIR**DVENAISEIKEDLEAEK**RSKERAEKAKRDLAEEIESLKMELLDTGNTSEEQQTILRKKEVELQNIKKSLDDETKRAEAEIQEIRKKAAVSIENLNNQLETSNKSKVAIEKAK**LQLQEELNDANNELSILR**SSKAESEKLKKNTEQQLSESLNRLAEVEEYKLDIETKLKKQTEVCDKLALSLESSESKLSQITKSEISLKSQLEDLQKNFEEETRLKLAAQTSLRQVIGEISQIKDQLEEEQQMKENLEKHVQILQTQMQEVKKKVEDDANQLLELDDSRKKIMREREELQIRLEEALNQVDRSEKAKRIIQAELQDAYHALEGQKNDQTQSDRKLKKMESQVNEIMNLNKKYLSEKEILEKENREKETRIMQLQREITANEDKLIELENSKSLLTRQLNELVSNKDDVGKNIGSLEKVKIQLETDLDNCKNTIEELEEENATLQMNKERIEMQLTATKAQLDRELSSKDEMHDEQFKILNKKLRDTEATLEEEQKQRTSLASLKKKLEIENVDLMNQLSESEKIKEDLSRQIKKHQGTSINSQREMQEALNAKNEALKQIKELEKRLRTADNSINQLTEDLSQLERVKRTIITERDDALEEIATLTVNKDTAIADKKRIESNLSALNMDYEELQLLLAELEDKNKK**ITNQLEQVQNELNVER**QNVTRLENQKSTAERLNKELHEKIEELEVEAGKKYKTTVAAQQNKIQSLDAQLDAKSEEINQANRNNKKLERKLKEIMALMVDEQKRADNAIEQIEKATGRYKKAERSLQMEQENNSALTTQNRRLQRDLEEVNETKEARDQEIKVLKTKIERLERRVRTGGRPNRGDAVSDDGLSGDGESLTDSGLGAVQQD | SMU15003136 | 86.87 | 1973 | 258 | putative myosin heavy chain |
| comp131819_c0_seq1 | 2 | [K].TPNFDYLISQGTR.[I]  [R].LTASHLWPLTDAPIR.[G] | MQNIQMRLCILFLFTGLCFIATAILLFQLIPEKWLVDRNQVLLISMDGFRHDYIEKVKANGGK**TPNFDYLISQGTR**IMRSQNAFPTITLPNHQTIVTGLYPENHGIVLNTFYDKNETFDMNNQNNLNDKKWFEKWPEPIWVTLEKLGR**LTASHLWPLTDAPIR**GKLPFFQESQYTLLDNPPNYFPFMKRVEDVVNWLSNTRVHIDFSMLYFPEPDETGHGYGPNDQHVFDVVMKLDAVMGHLIQLLKSKGLWDRVNIILTADHGMSENSFSRQIPLDTYVNPSWYSYTLLTPVGALYPVKGKEQLVYNALKNKNPHLQVFWREEVPRSLNYNIDNGRIAPLILIAENKWAISHKASNSTTIYGNHGYNNSEPDVQPFFIAQGPNIRSNYTIPFGYSVDIYPLMCYLLGIKPNPNNGSFERIKSLIEPKSIRNYDNTILYMKYDIWITWTCIAIFGISFIIIIVVHIQSVRKKRLSTKLKSAFAYSKIDSFY | SMU15000136 | 78.94 | 470 | 95 | putative pyrophosphatase phosphodiesterase family member |
| comp141188_c2_seq3 | 2 | [K].LISDVQEILLSNPTDMK.[S]  [K].SSELSGLLNELR.[K] | MALDIDLDIFRSKLADCKDRSQNSHFGVTSRALVAEEHRQKLILQELRELESKFKFRDDRTSKTAAQISEIKDEIDSNISAMKYLGGISNMENLYSELTGNIGACVVHHSVNEYIPVNTLASKPLSSNINECWNYITKLSQLSQVHLRNASHYHQFYHDLNEISALLEKRWKIIANQISNFEPKGTIKESEQLANYLKDHLSYFMHLRNRIDNLQSESKRIVPIHLRQDPVSQNKLVGHSLCSYSSPEISVKDHEEVFILNNSHPTTWKIQNSRGEIGEIPSVCIWIPGPDKLSSDKAISLQTQLIKNWEQCLNRFSIVLIDHYISLFERLLTDPVVCVNRSDPFNK**LISDVQEILLSNPTDMK**-**SSELSGLLNELR**KKLMLKHSEDSIATHLYTESETIKMHTPLIKYIDHLKNLRSLRDSIQFNDYHQHNRETKEKLYNSNLESLNKLYSENKHDLNKLLQSVDSIPNETYRHRHSQEIVHNSRSRDHINRDHRQFLSSSDLNHSHNYHLKSNHPEHHFAYKHDHLTPDTYSPSVSLTTSDIQLNRMNSPDYREFSNVRSIDNLTRNDNEEFHEYDDKLKSRTLPLVLNSEEIKPKSHHKKKKTWKVDSQTQLGVTVRDSKIQCNPTMYDESTFIEDEIFSTTDSSVTSSKQWSKSKAQKRYHLDAQTQIGVL | SMU15033813 | 68.08 | 683 | 206 | unknown |
| comp145670_c2_seq3 | 2 | [R].LIDGDSSSSGLVQIK.[Y]  [K].SALYYESNSGNR.[N] | MSPNYMPNNLALTDYMRYWNTTDNLRCVYMISSRKQNSFTIKITALTYFWYNIWINNQNVNGLTKITFSGDQRVSVVFEAYYYWYYSWYYYNSLSGFTGAVDLAYDVLNLLPVDNVPFRGVPQISYQGVNYSVQYESLSPLDAQVMCLTLGAVGYKSQKPSLNFTDRKLVSPSYQCLGPEKLLFHCLDLDVQKLFYKEFYNAPKYTTVLTCSEKFKLR**LIDGDSSSSGLVQIK**YGGIWSYMCTDNMDSSAADVICGELGYK**SALYYESNSGNR**NSSNYYNFPDGEYYKYRYGTVMNNIRCNSETKLKNCYYQTSNNYYNYYYYF | SMU15005691 | 43.61 | 321 | 173 | putative protease serine 12 neurotrypsin motopsin |
| comp146596_c0_seq1 | 2 | [K].SNQAYTEALDLSDELGATHPIR.[L]  [K].AISELDSLTEEDSYK.[D] | MTERDENVYKAKLSEQAERYDEMVKSIKLVAESGVELTVEERNLLSVAYKNVIGSRRSSWRILSSIEQKEESKATNTNRIEIARNYRKQVEKELSGICGEVIKILDDHLIKTATTTDSKVFYLKMKGDYYRYNAEFSIEKDRQEIAEK**SNQAYTEALDLSDELGATHPIR**LGLALNYSVFFYEIMGSSEKACELAKKAFDK**AISELDSLTEEDSYK**DSTLIMQLLRDNLTLWTADDDDQKNEGD | SMU15025275 | 93.36 | 241 | 16 | putative 14-3-3 protein |
| comp141858_c0_seq6 | 2 | [R].AVLVDLEPGTMDSVR.[A]  [K].EVDEQMLNVQNK.[N] | MREIVHLQAGQCGNQIGSKFWEVISDEHGIDPTGTYHGDSDLQLERINVYYNEATGGKYVPR**AVLVDLEPGTMDSVR**AGPFGQIFRPDNFIFGQSGAGNNWAKGHYTEGAELVDSVLDIVRKEAESCDCLQGFQLTHSLGGGTGSGMGTLLISKIREEYPDRVMLTFSVVPSPKVSDTVVEPYNATLSVHQLVENTDETFCIDNEALYDICFRTLKLTTPTYGDLNHLVSATMSGVTTCLRFPGQLNADLRKLAVNMVPFPRLHFFMPGFAPLTSRGSQQYRALTVPELTQQIFDAKNMMAACDPRHGRYLTVAAIFRGRMSMK**EVDEQMLNVQNK**NSSYFVEWIPNNVKTAVCDIPPRGLKMSSTFIGNSTAIQELFKRVSEQFTAMFRRKAFLHWYTGEGMDEMEFTEAESNMNDLVSEYQQYQDATAEEEGEAEDEEEAEQ | SMU15002375 | 97.52 | 443 | 10 | putative tubulin beta |
| comp135193_c0_seq1 | 2 | [R].EQTGEDLPAGVIQELNEQEK.[R]  [R].ALYICDGGEDEASEIITK.[L] | MHQLIWEGDNEIPQEVHEVVIPHLGHMPICKGDYHYLPKAVQGFIAKWALICKPR**ALYICDGGEDEASEIITK**LKERGTLHALTKLENCYICRTDPDDVARVESKTFISTKDKHMTVPHVKEGSTGILGQWMAPESLSEEVAQRLRGCMAGRMMYIIPFSMGPIGSPLSKIGIQLTDSNYVVLSMRVMTRVSPLVWHYLGDKAFVKCVHSVGCPRPHQRKVVNHWPCNPEKTLIAHLPDERLIISYGSGYGGNSLLGKKCFALRIAGRIAYDEGWMAEHMLIMSVTNPKGEEKFIAAAFPSACGKTNMAMLEPSLPGWKVQVVGDDIAWMRFDEHGELRAINPEAGFFGVAPGTNYKTNPNAMKTFQKNSIFTNVAETSDGSYYWEGLEDEIDKNLEITSWLGVKMRVGDKSKGLAAHANSRFCCPASQCPIIHPKWEDPKGVPISAIVFGGRRPEGIPLVMQAFNWKHGVMLGASLKSEATAAAEFKGKQIMHDPMAMRPFMGYNFGKYLQHWLDLEKPGRKMPLIFHVNWFRLNDKGKFVWPGFGDNIRVIDWMCRRVNGEDIAEPSPIGLIPKHGTINVTGLDAKWEEMFALPKTYLAEDIQETVKFLR**EQTGEDLPAGVIQELNEQEK**RIHNM | SMU15002770 | 91.96 | 634 | 51 | putative phosphoenolpyruvate carboxykinase |
| comp86205_c0_seq1 | 2 | [R].VFDKDGNGFISAAELR.[H]  [K].EAFSLFDKDGDGTITTK.[E] | MADQLTEEQIAEFK**EAFSLFDKDGDGTITTK**ELGTVMRSLGQNPTEAELQDMINEVDADGNGTIDFPEFLTMMARKMKDTDSEEEIREAFR**VFDKDGNGFISAAELR**HVMTNLGEKLTDEEVDEMIREADIDGDGQVNYEEFVTMMTTK | SMU15039048 | 100.00 | 149 | 0 | putative protein CMD-1 (calmodulin) |
| comp142073_c0_seq1 | 2 | [K].VILSFDNGIICETYENVK.[I]  [K].LIENNQIQYNK.[V] | MMKLNFFLILLLSIVRNYCLCNENEQWTSENEYSNYQVETLQKITDWDCWYKDYYLKVSHCPSKTLKSKEKIKCKQYKDCKRYCSSVYGIEQCRVRCQSKKTDLSKTIKVIYRVRKDGECKDSVICVYRTKCQGLWPKYIHQGCYYTGEFKSWWLKHKKEYAKPRIYGQIQPSGKIYNCIINNARFPLINPSLETTVQFRMSDNKKLFYKIGQKWFKAKFVVGNLFFWIGKLWVPVRFSRKHQYWKIGDKWIQVIVLRNTVYSNINSSWIPLKVMFGKKYFKIGKRWLNLKITNNKIHYKFGGKWIEIKILQNNLYNKINGVWYKVKFHKNFNLIKIGQSYRKIKSIHEIIYMKLGKRWFEFTVENKRYQIRFDKKWVEIIFDKGLVFFLIDNNKYQIRFKFYSMFIELNKKWFQIEFQKEKLFLKTNKIWLHILLKENHFYIKYNGKERKLIVDENRNIFYKSSLKDKLLSLDEKEIKSGNSFPIDQFIKKENSFSLKYFSGYWLPFKIEKGESLFFISGKWLNLKNENHKLFYKYQEQWIEVCFIGKNEFNKIQGHWIQVKRDENRLFYFDSKFWHQIQLTGKYVAIKINDSWKSFAFVNGKKFIRLGNKFSEIKFNSENQNEFHLKIGGKWHNLKMENRNFFIEFNEKWTRVEFYSHRSMNILFDNNWLSVTFKNKKLYVKNENCWLETIFKTNKIFMKIGDTLTEMKISENKDFKVKINGHFYK**LIENNQIQYNK**VILKISFINEKLHAFINGNWLRLRIDKENLMVKVIDSWIEAEIDENKQISVKVNNEK**VILSFDNGIICETYENVK**IPISIRFKQLYYNLDGKWIKIYLKDNLRYFKENGNLVILDRDFLKNKTRKYKLKLMLKCTLLSDNIKELTNFGRSEFKRNIQRNKHENMRYYQTIDMLKKPRQKSKQEII | SMU15038409 | 53.64 | 934 | 421 | unknown |
