# Supplementary material for: Planarian regeneration in space: Persistent anatomical, behavioral, and bacteriological changes induced by space travel
Source: Regeneration (Oxf). 2017 Jun 13;4(2):85–102. doi: 10.1002/reg2.79 (PMC5469732; doi:10.1002/reg2.79)
Supplement: Supplementary file 6 — Supplemental Table 2: Worm colony growth after space mission. [file REG2-4-85-s006.docx]

**Supplemental Table 2: Worm colony growth after space mission.** The number of worms before and after one month in a sealed tube, either while traveling to space and back, or left on Earth, together with number of worms after additional two months on Earth. Worm colonies which have traveled to space, all have shown slightly reduced rate in colony size growth, compared to their Earth counterparts. (See Supplemental Figure 3.) Note that the colony from 10 whole worms left of Earth did not survive the duration of the additional two months on Earth for unknown reason.

| Start n | 4 | 5 | 6 | 8 | 10 |
| --- | --- | --- | --- | --- | --- |
| 1 month  on Earth | 4 | 5 | 6 | 8 | 10 |
| & +2 months  on Earth | 39 | 38 | 80 | 60 | (-) |
| 1 month  in Space | 7 | 7 | 10 | 14 | 13 |
| & +2 months  on Earth | 35 | 35 | 42 | 44 | 49 |
| in 3 months | 0.90x | 0.92x | 0.53x | 0.73x | (-) |
